# Membrane progesterone receptor induces meiosis in *Xenopus* oocytes through endocytosis into signaling endosomes and interaction with APPL1 and Akt2

**DOI:** 10.1371/journal.pbio.3000901

**Published:** 2020-11-02

**Authors:** Nancy Nader, Maya Dib, Rawad Hodeify, Raphael Courjaret, Asha Elmi, Ayat S. Hammad, Raja Dey, Xin-Yun Huang, Khaled Machaca

**Affiliations:** 1 Department of Physiology and Biophysics, Weill Cornell Medicine Qatar, Education City, Qatar Foundation, Doha, Qatar; 2 Calcium Signaling Group, Weill Cornell Medicine Qatar; 3 College of Health and Life Science, Hamad bin Khalifa University, Doha, Qatar; 4 Department of Physiology and Biophysics, Weill Cornell Medicine, New York, United States of America; Osaka University, JAPAN

## Abstract

The steroid hormone progesterone (P4) mediates many physiological processes through either nuclear receptors that modulate gene expression or membrane P4 receptors (mPRs) that mediate nongenomic signaling. mPR signaling remains poorly understood. Here we show that the topology of mPRβ is similar to adiponectin receptors and opposite to that of G-protein-coupled receptors (GPCRs). Using *Xenopus* oocyte meiosis as a well-established physiological readout of nongenomic P4 signaling, we demonstrate that mPRβ signaling requires the adaptor protein APPL1 and the kinase Akt2. We further show that P4 induces clathrin-dependent endocytosis of mPRβ into signaling endosome, where mPR interacts transiently with APPL1 and Akt2 to induce meiosis. Our findings outline the early steps involved in mPR signaling and expand the spectrum of mPR signaling through the multitude of pathways involving APPL1.

## Abstract 2

The steroid hormone progesterone mediates many physiological processes through either nuclear receptors that modulate gene expression, or membrane progesterone receptors (mPRs) that mediate non-genomic signaling. This study shows that non-genomic mPRβ signaling progresses through clathrin-dependent endocytosis into signaling endosomes where it interacts with and activates APPL1 and Akt2 to induce oocyte meiosis.

## Introduction

Progesterone (P4) is an essential steroid hormone that mediates many physiological functions, including female reproduction, sperm activation, modulation of the immune system, neuroprotection, and neurogenesis [1–3]. P4 mediates its action through 2 types of signaling: genomic (classical) signaling through nuclear P4 receptors (PRs) that modulates gene transcription, and/or rapid nongenomic (nonclassical) signaling via membrane progesterone receptors (mPRs) among other effectors [2, 4]. Several lines of evidence support an important role for nongenomic P4 signaling, including P4-dependent events in cells lacking nuclear PRs; the ability to mediate P4 signaling using P4 coupled to bovine serum albumin (BSA), which cannot permeate the plasma membrane (PM) and activate PR; and rapid P4-dependent signaling on the order of seconds to minutes that is inconsistent with gene expression [2, 3].

mPR was first cloned from fish ovaries [5, 6] and belongs to the progesterone and adiponectin (AdipoQ) receptor family (PAQRs) that consists of 11 receptors with 5 members activated by P4: PAQR5 (mPRγ), PAQR6 (mPRδ), PAQR7 (mPRα), PAQR8 (mPRβ), and PAQR9 (mPRϵ) [4, 7]. Although PAQRs share a similar 7-transmembrane domain topology with G-protein-coupled receptors (GPCRs), the signal transduction pathway downstream of mPR remains poorly defined and controversial with evidence either supporting or refuting a role for heterotrimeric G-proteins [3, 4, 8, 9]. Although the topology of mPRs has not been assessed experimentally, bioinformatics approaches argue that mPRs have a similar topology to GPCRs [6, 7]. However, this conclusion is not widely accepted as other predictions suggest an intracellular N-terminus and little sequence homology to GPCRs [4, 7]. Furthermore, several studies argue that mPRs couple to heterotrimeric G-proteins, primarily Gα_i_ with some suggesting a role for Gα_s_ [4]. There is also evidence from different systems for crosstalk between mPR and PR signaling [3].

*Xenopus* oocyte maturation represents one of the oldest and most extensively studied nongenomic P4 physiological responses, because the oocyte is transcriptionally silent and BSA-coupled P4 induces oocyte maturation. Although a role for the classical PRs cannot be completely ruled out [10, 11], the response to release meiotic arrest is primarily through mPRβ [12–14].

mPRβ, through a complex signal transduction pathway that involves the activation of the mitogen-activated protein kinase (MAPK) cascade, Polo-like kinase 1 (Plk1), and the phosphatase Cdc25C, activates maturation promoting factor (MPF or cyclin-dependent kinase 1 (Cdk1), the master kinase that commits the oocyte to meiosis [15, 16]. As with other systems, however, the early signaling events downstream of mPRβ remain obscure despite much effort over the past 4–5 decades. Furthermore, mPR in the frog oocyte does not signal through Gα_i_ because pertussis toxin does not block maturation [17, 18]. Recent data also argue against mPR mediating its signaling by acting on the adenylate cyclase-cAMP pathway [19]. Upstream of the MAPK cascade, P4 triggers polyadenylation and translation of Mos, an oocyte-specific MAPK kinase activator [15, 16]. However, the signaling events upstream of Plk1 are still unknown.

Here we show that mPRβ has a cytosolic N-terminus and an extracellular C-terminus, a topology that matches the AdipoR and is opposite to that of GPCRs. We further demonstrate that mPRβ signals through APPL1 (Adapter protein containing Pleckstrin homology domain, Phosphotyrosine binding domain and Leucine zipper motif 1) and its interacting partner, the serine/threonine kinase Akt2 (also known as protein kinase B). Interestingly, mPRβ signaling requires endocytosis through the clathrin pathway, a process that depends on APPL1. mPR associates with APPL1 and Akt2 into signaling endosomes minutes after P4 addition. Collectively, these data elucidate the early steps downstream of mPRβ and uncover a novel reliance on clathrin-dependent endocytosis and APPL1-Akt2.

## Results

### mPRβ topology

**Fig 1:**
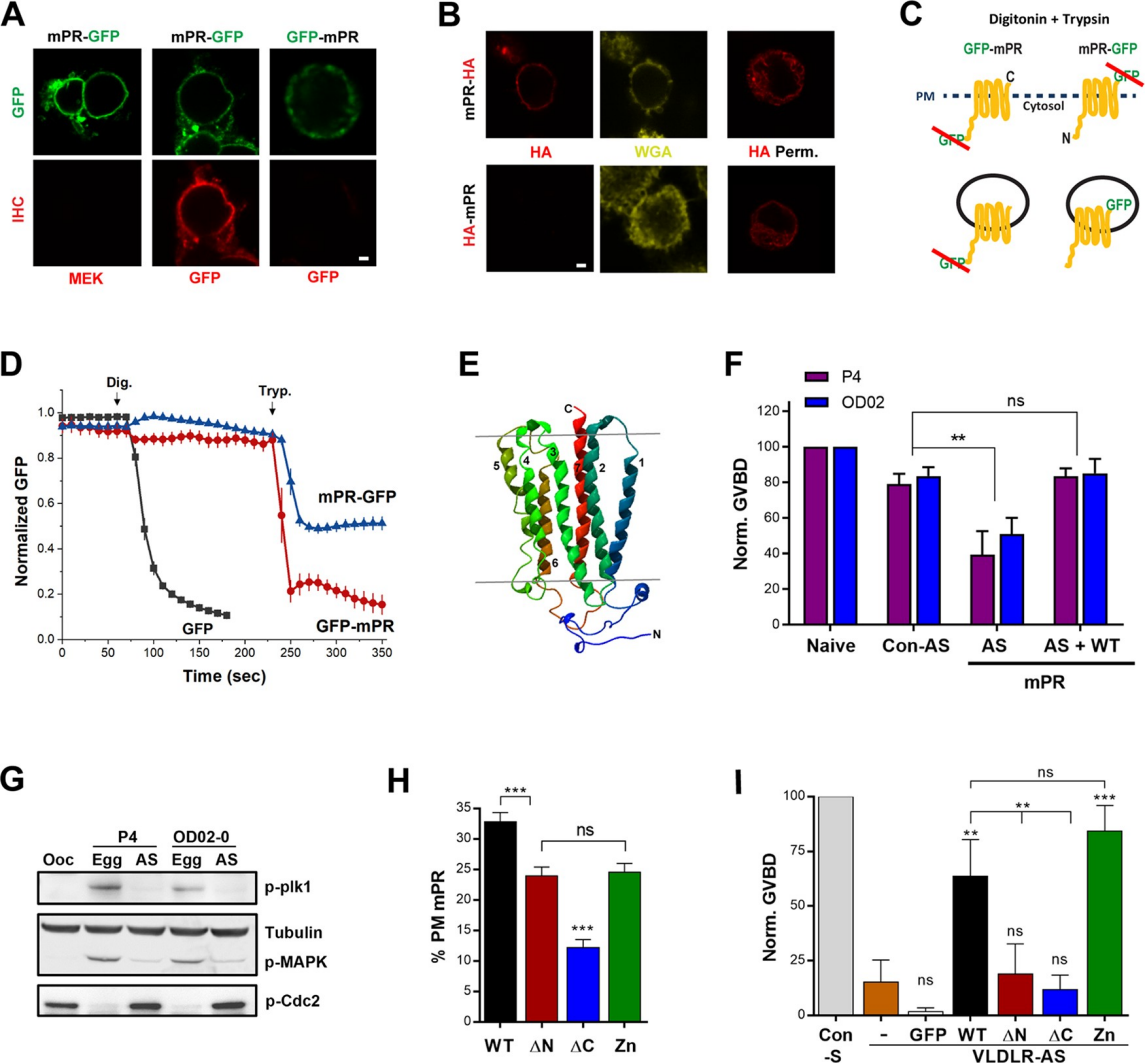
mPRβ has an opposite topology to GPCRs and is required for oocyte meiosis. (A) Nonpermeabilized CHO cells overexpressing mPR-GFP or GFP-mPR stained with anti-GFP or anti-MEK antibodies. Scale bar 2 μm. (B) Immunostaining of CHO cells overexpressing mPR-HA or HA-mPR in nonpermeabilized or permeabilized (HA Perm) conditions. PM stained with WGA. Scale bar 2 μm. (C) Schematic representation of the FPP assay. (D) Time course of GFP fluorescence in cells transfected as indicated and treated with Dig then Tryp at the indicated time points (mean ± SEM; *n =* 8–11 cells). (E) 3D model of *Xenopus* mPRβ. (F) Oocyte maturation in response to P4 or OD 0–02 in naive oocytes, oocytes injected with Con-AS, oocytes injected with specific mPR-AS, and oocytes injected with antisense oligos and expressing WT mPR-GFP (50 ng/oocyte) (AS + WT) (mean ± SEM; *n =* 3–4 donor females). (G) Activation state of MAPK and Plk for the treatments in panel (F). For the AS treatments, oocytes that did not mature (no white spot) in response to P4 were collected when the control group has reached maximal maturation levels. (H) Percent of the total mPR population at the PM for full-length mPR (WT) and the different mutants as indicated (mean ± SEM; 15–23 oocytes per condition, from 3 donor females). (I) GVBD rescue using the mPR mutants (20 ng/oocyte) following VLDLR-AS or sense oligos injection (Con-S). GFP: GFP RNA injection as a control (mean ± SEM; *n =* 3 donor females). ** *p* < 0.01, *** *p* < 0.001. Refer to S1 Data file. AS, injected with mPR antisense oligos; Con-AS, control antisense oligos; Dig, digitonin; Egg, eggs matured with P4; FPP, fluorescence protease protection; GFP, green fluorescent protein; GVBD, germinal vesicle breakdown; HA, hemagglutinin tag; MAPK, mitogen-activated protein kinase; mPR, membrane progesterone receptor; MEK, mitogen-activated protein kinase kinase; ns, not significant; Ooc, untreated oocyte; P4, progesterone; Plk1, Polo-like kinase 1; PM, plasma membrane; VLDLR, very-low-density lipoprotein receptor; VLDLR-AS, VLDLR antisense knockdown; Tryp, trypsin; WGA, wheat germ agglutinin; WT, wild type.

To better define the immediate signaling pathway downstream of mPRβ, referred to here as mPR for simplicity, we first studied its topology. We generated mPR clones tagged with green fluorescent protein (GFP) at either their N- (GFP-mPR) or C- (mPR-GFP) terminus and overexpressed them in Chinese hamster ovary (CHO) cells. We immunostained for GFP under nonpermeabilized conditions, as confirmed by staining for the cytosolic protein mitogen-activated protein kinase kinase (MEK) (Fig 1A), and readily detected the C-terminally tagged mPR, showing extracellular GFP localization (Fig 1A). In contrast, no staining was apparent for N-terminally tagged GFP-mPR arguing for intracellular localization (Fig 1A). However, under these conditions, GFP-mPR did not traffic properly to the cell membrane based on the diffuse GFP signal (Fig 1A). The impaired trafficking of GFP-mPR could be due to the large GFP tag, so we replaced it with the smaller HA-tag (8 residues).

As with GFP-tagged mPR, the C-terminal HA-tag was extracellular (Fig 1B), whereas the N-terminally tagged HA-mPR was protected under nonpermeabilized conditions (Fig 1B), arguing for cytoplasmic localization. In permeabilized cells, both HA-mPR and mPR-HA exhibit both cortical and diffuse intracellular staining (Fig 1B), raising the possibility that the N-terminal tagged mPR does not traffic properly. Therefore, to confirm the cytosolic localization of the mPR N-terminus, we used the fluorescence protease protection (FPP) assay, which permits the determination of membrane protein topology in living cells. FPP employs the spatially confined action of trypsin following digitonin treatment to permeabilize only the PM while leaving intracellular organelles intact [20] (Fig 1C). Treating cells expressing GFP alone with digitonin leads to loss of the fluorescence signal showing effective permeabilization (Fig 1D). In cells expressing mPR-GFP, trypsin addition results in loss of 50% of the signal, consistent with the extracellular localization of the GFP tag (Fig 1C and 1D). In this case, GFP is protected from trypsin within intracellular vesicles/organelles because of its luminal position (Fig 1C). In contrast, the GFP-mPR signal is completely lost following trypsin treatment, confirming the cytoplasmic localization of the mPR N-terminus (Fig 1D). Combined, these data demonstrate that mPR has an intracellular N-terminus and an extracellular C-terminus, a similar topology to adiponectin receptors (AdipoRs) [21, 22] and opposite to GPCRs [23]. This finding is consistent with the sequence homology that places mPR within the PAQR family, that includes the well-studied AdipoR1 and AdipoR2 receptors.

Given the AdipoR homology, we modeled the 3D structure of mPR based on the solved crystal structures of AdipoRs (Fig 1E). Aligning mPR with human and *Xenopus* AdipoRs shows sequence conservation (S1A Fig). We first generated a homology model of the *Xenopus* AdipoR based on its human orthologue and used that structure to model mPR shown in either the side view (Fig 1E) or an orthogonal (extracellular) view (S1B Fig). Superimposing the 3D structure of mPR on the human AdipoR2 displays significant alignment, with 7 transmembrane domains (S1C Fig). Together with the topology experiments, these modeling studies support the conclusion that mPR has the same topology as AdipoRs.

### mPR is required to release oocyte meiotic arrest

We then used the *Xenopus laevis* oocyte as an experimental model to define signaling events downstream of mPR. *Xenopus* oocytes arrest at prophase of meiosis I and must undergo a maturation period before they become fertilization competent and able to support embryonic development. P4 results in the dissolution of the nuclear envelope (referred to as germinal vesicle breakdown [GVBD]), chromosome condensation, and the arrest in metaphase of meiosis II [15, 24]. The tetraploid *Xenopus* genome contains 2 subgenomes referred to as L (Long) and S (Short), originating from distinct diploid progenitors [25, 26]. Thus, the mPR gene has 2 paralogues in the genome (L and S) that are highly conserved (S1D Fig). To test the role of mPR in oocyte maturation, we knocked down endogenous mPR mRNA using antisense oligos (S1E Fig). We were unable to test knockdown of endogenous mPR expression as antibodies for mPR are not available. We therefore overexpressed mPR-GFP and show that the antisense effectively blocks its expression (S1F Fig). Functionally, knockdown of mPR (mPR-KD) inhibited P4-dependent maturation as marked by GVBD (Fig 1F). This inhibition of GVBD was reversed by expressing wild-type (WT) mPR and control antisense oligos had no significant effect on GVBD (Fig 1F). This shows the specificity of the antisense mPR knockdown. The GVBD block following mPR-KD was confirmed biochemically by the inhibition of MAPK and Plk1, both activated by phosphorylation (Fig 1G), as well as MPF that is activated by dephosphorylation of its catalytic subunit (Cdc2) (Fig 1G) [27]. Similarly, mPR knockdown blocked maturation in response to the mPR specific agonist OD 02–0 [28] (Fig 1F and 1G), arguing against any involvement of nuclear PRs. The mPR-KD inhibition of maturation in response to OD 02–0 was also rescued following expression of mPR and not affected by control antisense (Fig 1F). These results confirm the essential role of mPR in inducing oocyte maturation in response to P4 as previously proposed [12].

To further dissect the domain requirements for mPR function, we generated 3 mutants: mPR-ΔN that lacks the cytosolic N-terminal domain, mPR-ΔC that lacks the last 12 residues, and mPR-Zn with the conserved Zn^2+^ binding residues, based on the AdipoR alignment, mutated to alanine (S1D Fig). First, we assessed the trafficking of the different mutants to the PM, in oocytes co-expressing the endoplasmic reticulum (ER) marker KDEL-mCherry and stained with the PM marker wheat germ agglutinin (WGA) (S2A Fig). Both the ΔN and Zn mutants trafficked normally to the PM, albeit with an approximately 20% decrease in PM residence as compared with mPR WT (Fig 1H and S2A Fig). In contrast, the ΔC mutant was mostly intracellular and localized to the ER (Fig 1H and S2A Fig).

To test the functionality of these mutants, we relied on our recent identification of the very-low-density lipoprotein receptor (VLDLR) as an mPR chaperone required for its trafficking to the PM [29]. VLDLR knockdown inhibits GVBD, a phenotype that is rescued by overexpressing mPR-GFP but not GFP alone (Fig 1I) [29]. Expressing the Zn mutant also rescues VLDLR knockdown (Fig 1I) but not the ΔN nor ΔC mutants (Fig 1I). Together the trafficking and functional data argue that the cytosolic N-terminal domain of mPR is required for its signaling, whereas the putative Zn^2+^ coordinating residues are dispensable. The ΔC mutant does not rescue GVBD following VLDLR knockdown (Fig 1I) and does not co-localize with VLDLR (S2B Fig), arguing that the terminal 12 mPR residues are required for its interaction with VLDLR and as such, trafficking to the PM. Combined, these results show that mPR is essential for P4-mediated oocyte maturation, with its N-terminus required for signaling, whereas its C-terminus required for PM trafficking.

### APPL1 is essential for P4-mPR-induced resumption of oocyte meiosis

The structural and topological homology between AdipoR and mPR (Fig 1E and S1A–S1C Fig) suggests that these 2 receptors signal via similar pathways, and especially that in yeast, both receptor families couple to the same signal transduction pathway [30]. A critical player downstream of AdipoRs is APPL1 [31]. Therefore, we investigated the role of APPL1 in mPR signaling.

**Fig 2:**
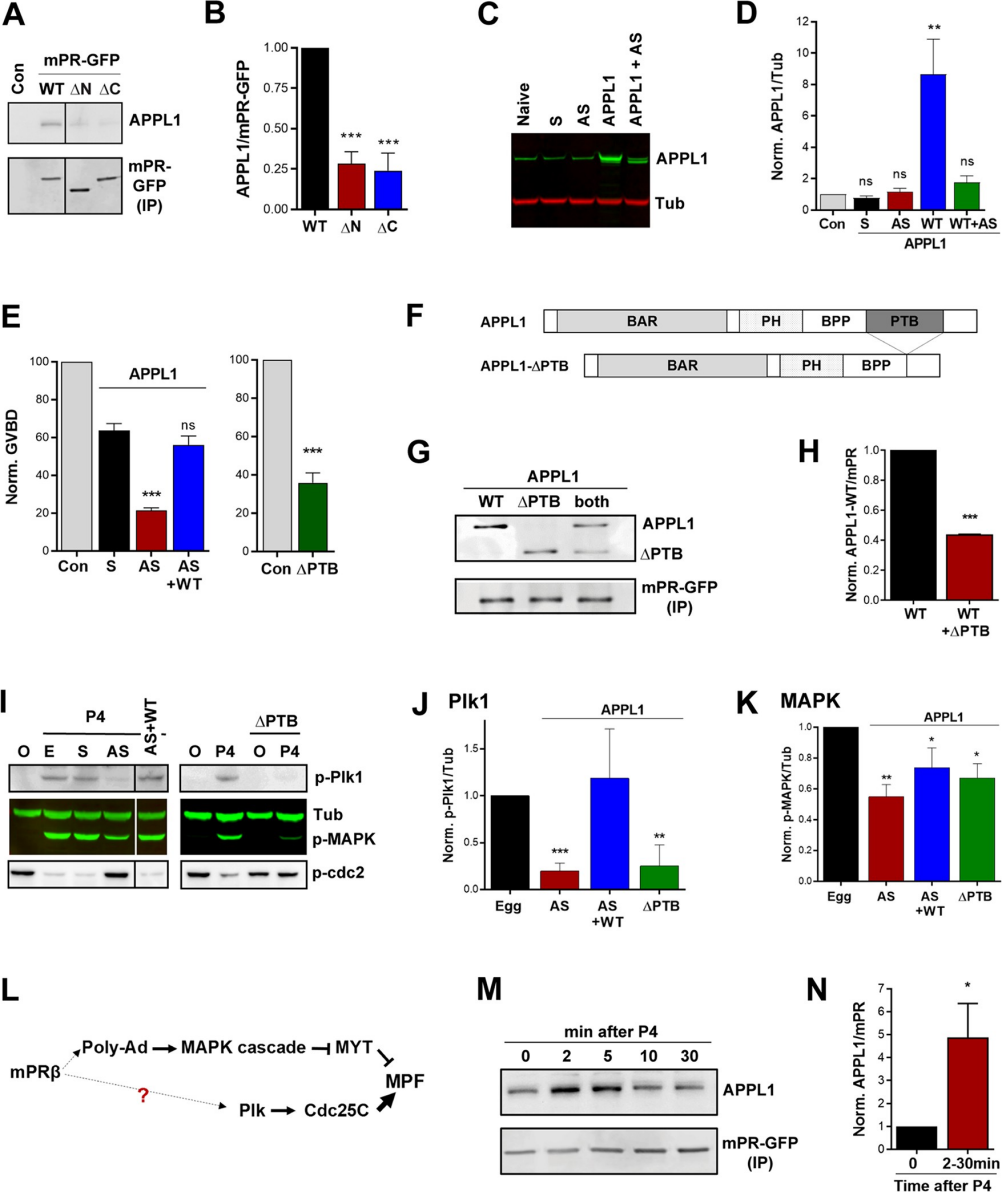
APPL1 is essential for P4-mPR-induced oocyte meiosis. (A) IP of mPR-GFP from untreated oocytes (Con) and oocytes expressing mPR-GFP (WT) (20 ng/oocyte), the ΔN or ΔC mutants (20 ng/oocyte) probed for APPL1 and GFP. (B) Quantification of the IP experiments as the ratio of APPL1/mPR-GFP normalized to mPR-GFP WT expressing cells (mean ± SEM; *n =* 3). (C) WB analysis of APPL1 in untreated (naive), sense (S), antisense (AS) APPL1 oligos injected oocytes, in oocytes expressing APPL1.L (APPL1) (50 ng/oocyte), and in APPL1.L expressing oocytes co-injected with APPL1 antisense oligos (APPL1+AS). Tubulin is shown as a loading control. (D) Quantification of APPL1 expression normalized to tubulin for the conditions in panel (C) (mean ± SEM; *n =* 3). (E) Oocyte maturation measured as the levels of GVBD normalized to untreated oocytes (Con) in oocytes injected with APPL1 sense (S) or antisense oligos (AS), injected with APPL1 antisense oligos and APPL1 mRNA to overexpress APPL1 (AS + WT) (50 ng/oocyte) (left panel, mean ± SEM, *n =* 4 donor females), or injected APPL1-ΔPTB (ΔPTB) RNA (50 ng/oocyte) (right panel, mean ± SEM; *n =* 3 donor females). (F) Schematic of APPL1 full-length and APPL1-ΔPTB domains. (G) IP of mPR-GFP from oocytes expressing APPL1 full-length (WT) (20 ng/oocyte), APPL1-ΔPTB (ΔPTB) (20 ng/oocyte), or both proteins probed for APPL1 and GFP. (H) Quantification of the IP experiments in (G) as the ratio of APPL1/mPR-GFP normalized to mPR-GFP injected oocytes (mean ± SEM; *n =* 2). (I) Left Panel: WB of MAPK, Plk1, and Cdc2 phosphorylation from untreated oocytes (O), eggs matured with P4 (E), oocytes injected with APPL1 sense (S), antisense (AS) oligos, or antisense oligos and APPL1 RNA (AS+WT). Right Panel: WB of untreated oocytes (O) and P4 matured eggs (P4), and APPL1-ΔPTB injected oocytes untreated or P4 treated as indicated. Tub is shown as a loading control. For the AS and ΔPTB treatments with P4 immature oocytes with no white spot where collected at the end of the experiment when the control group has reached maximal GVBD levels. (J-K) Quantification of p-Plk1 as the ratio of p-Plk1/Tub (J) or p-MAPK as the ratio of p-MAPK/Tub (K) normalized to the ratios in untreated eggs (mean ± SEM; *n =* 3–5 donor females). (L) Current model for the signaling cascade downstream of mPR. (M) IP from oocytes overexpressing mPR-GFP (20 ng/oocyte) and treated with P4 (10^−5^ M) for the indicated times and probed for APPL1. (N) Quantification of the IP experiments in (M) as the ratio of APPL1/mPR-GFP normalized to untreated oocytes (time 0). The max response between 2 to 30 min from each donor frog was used for the analysis (2–30 minutes) (mean ± SEM; *n =* 5). *p < 0.05, **p < 0.01, *** *p* < 0.001. Refer to S1_Data file. APPL, adapter protein containing Pleckstrin homology domain, Phosphotyrosine binding domain and Leucine zipper motif 1; AS, antisense; BAR, bin, amphiphysin and Rvs domain; GFP, green fluorescent protein; GVBD, germinal vesicle breakdown; HA,; IP, immmunoprecipitation; MAPK, mitogen-activated protein kinase; mPR, membrane progesterone receptor; MEK, mitogen-activated protein kinase kinase; ns, not significant; P4, progesterone; Plk1, Polo-like kinase 1; PTB, phosphotyrosine-binding domain; Tub, tubulin; WB, western blot; WT, wild type.

Immunoprecipitation experiments show that endogenous APPL1 interacts with overexpressed full-length mPR and fails to do so with either the ΔN or ΔC mPR mutants (Fig 2A and 2B), both of which are not effective in inducing maturation (Fig 1I). To test the role of APPL1 in mPR signaling, we knocked down APPL1.L expression because it has close homology to human APPL1, whereas APPL1.S was more divergent (S2C Fig). APPL1.L antisense oligos effectively knocked down endogenous APPL1.L mRNA levels and to a much lesser extent, APPL1.S levels (S3A Fig). This results in loss of co-immunoprecipitation with mPR-GFP (S3B Fig), arguing that APPL1.L is the main physiological mPR partner. The APPL1 antibody used was raised against full-length human APPL1 [32] and is predicted to recognize both APPL1.L and APPL1.S given their sequence conservation (S2C Fig), which would explain the lack of detectable loss in total endogenous APPL1 (Fig 2C and 2D). On the other hand, overexpression of APPL1.L was blocked by the antisense (Fig 2C and 2D).

Knockdown of APPL1.L resulted in significant inhibition of P4-induced oocyte maturation (Fig 2E). This inhibition was specifically due to the loss of APPL1.L as it was rescued by overexpressing APPL1.L (Fig 2E). To further confirm the role of APPL1 in mediating mPR signaling, we used a dominant-negative approach by deleting the PTB domain of APPL1 (Fig 2F), which is known to bind AdipoR1 and the downstream effector Akt2 [31, 33]. Overexpressing APPL1-ΔPTB (ΔPTB) effectively blocks oocyte maturation but to a lesser extent than APPL1 knockdown (Fig 2E). APPL1-ΔPTB interacts with mPR and competes with full-length APPL1 (Fig 2G and 2H). This suggests that APPL1-ΔPTB acts as a dominant-negative in the oocyte by competing with endogenous APPL1 while preventing the stimulation of downstream effectors.

We then tested the activation status of the signaling cascade downstream of P4 by assessing both arms of the pathway: MAPK and Plk1, which converge on MPF [16] (Fig 2L). Interestingly, APPL1 knockdown inhibits Plk1 phosphorylation noticeably more than MAPK phosphorylation (Fig 2I, 2J and 2K). Similarly, the dominant-negative ΔPTB mutant inhibits Plk1 activation primarily and MAPK to a lower extent (Fig 2I, 2J and 2K). Collectively, these results show that APPL1 plays a crucial role in signaling downstream of mPR to induce oocyte maturation by primarily activating the Plk1-Cdc25C arm of the pathway.

Because APPL1 is essential for P4-dependent oocyte maturation, we tested whether the interaction between APPL1 and mPR is regulated by P4. Pulldown experiments show that APPL1 and mPR interact at rest in oocytes (Fig 2A); however, this interaction is significantly enhanced following P4 treatment (Fig 2M). In the example shown in Fig 2M, the APPL1-mPR association transiently peaks 2–5 minutes after P4 addition. Although P4 invariably resulted in increased interaction between APPL1 and mPR, the timing of this interaction was variable from frog to frog, so we measured the peak increase between 2–30 minutes from different donor females and consistently observed an increased time-dependent interaction (Fig 2N). This variability is to be expected because oocyte maturation is an asynchronous process with oocytes from different females activated along a different time course, as well as oocytes from the same female activating asynchronously in response to P4.

### mPR-APPL1 signal through Akt2

**Fig 3:**
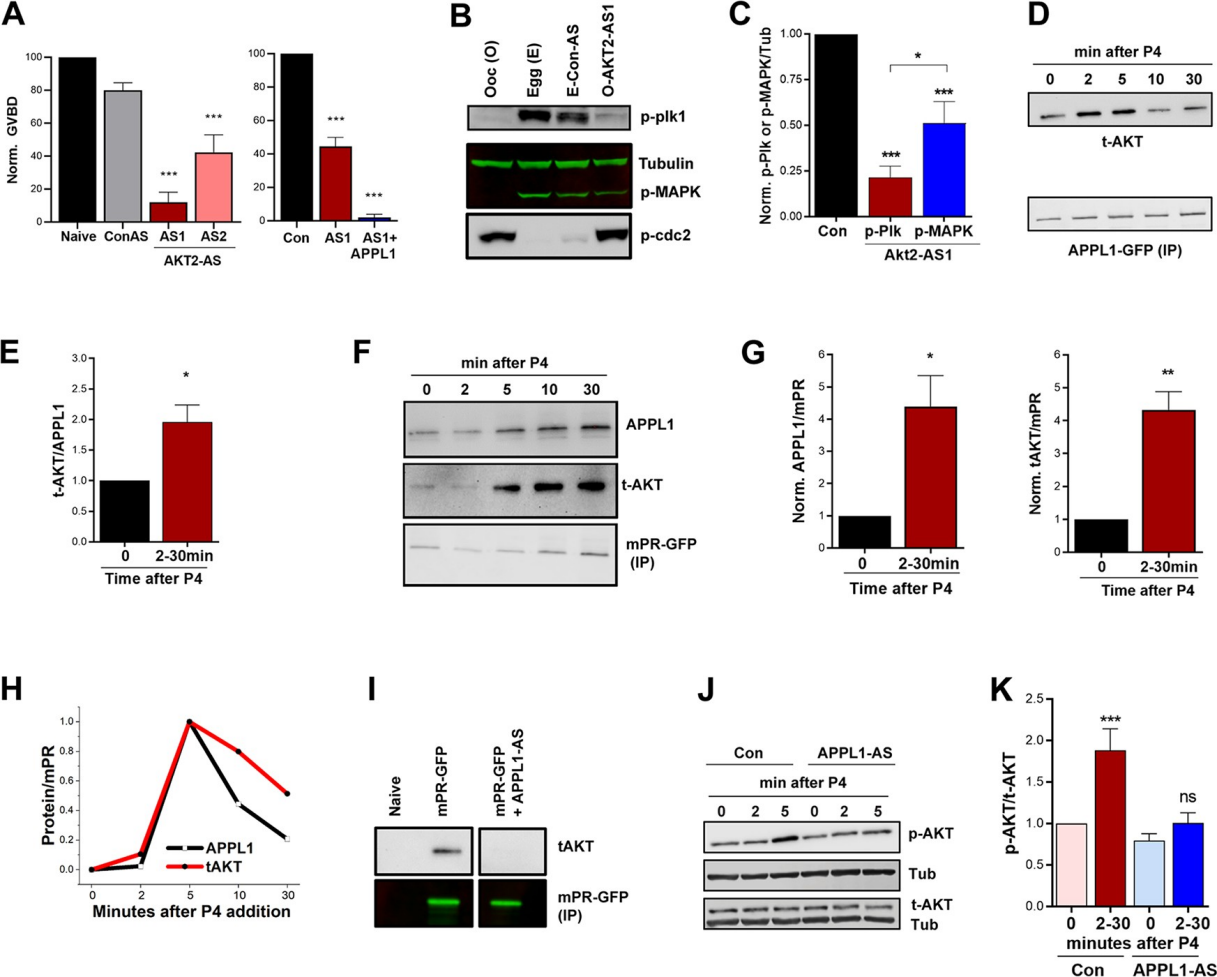
Akt2 is required for mPR signaling. (A) Oocyte maturation in response to P4 in untreated oocytes (Naive), oocytes injected with Con-AS oligos, oocyte injected with 2 different Akt2 antisense oligos (AS1 or AS2) (left panel), and in oocytes injected with Akt2-AS1 oligos with overexpression of APPL1 (1+APPL1) (50 ng/oocyte) (right panel). GVBD percentage is normalized to naive (mean ± SEM; *n =* 3–5 donor females). (B) Activation of the MAPK and Plk1 cascades in untreated oocytes (O), untreated eggs matured with P4 (E), eggs injected with Con-AS oligos and matured with P4 (E-Con-AS), or oocytes injected with Akt2 antisense oligos and treated with P4 (O-AKT-AS1). For the O-AKT-AS1 group immature oocyte with no white spot where collected when the control group has reached maximal GVBD levels. (C) Quantification of p-Plk1 and p-MAPK for the conditions in panel (B) as the ratio of p-Plk1 or p-MAPK to Tub, normalized to untreated eggs (Con) (mean ± SEM; *n =* 7). (D) IP over a P4 time course using overexpressed APPL1-GFP (20 ng/oocyte) as a bait and probed for t-AKT. (E) Quantification of Akt pulldown for the IP experiments in (D) as the ratio of t-AKT/APPL1-GFP. The maximal response between 2 to 30 minutes from each experiment is plotted (mean ± SEM; *n =* 5). (F) IP over a P4 time course using overexpressed mPR-GFP (20 ng/oocyte) as a bait and probed for APPL1 and total Akt. (G) Quantification of the IP experiments in (F) as the ratio of t-Akt/mPR-GFP. The max response between 2 to 30 minutes from each experiment is plotted (mean ± SEM; *n =* 5). (H) Normalized amounts of APPL1 and t-AKT that immunoprecipitate with mPR-GFP over time from a single experiment. (I) IP of mPR-GFP probed for t-AKT from oocytes expressing mPR-GFP without or with injection of APPL1 antisense oligos (mPR-GFP + APPL1-AS). (J) Western blot analysis of t-AKT and phospho-Akt S473 (p-AKT) over a P4 time course without (Con), or with APPL1 antisense oligos injection (APPL1-AS). Tub is used as a loading control. (K) Quantification of p-AKT for the conditions in panel (J). Maximal phospho/total Akt ratio between 2 to 30 minutes from each experiment is plotted. p-AKT and t-AKT levels were first normalized to Tub. Data are normalized to untreated oocytes (time 0) (mean ± SEM; *n =* 6 donor females). *p < 0.05, ** *p* < 0.01, ***p < 0.001. Refer to S1 Data file. APPL, adapter protein containing Pleckstrin homology domain, Phosphotyrosine binding domain and Leucine zipper motif 1; AS, antisense; Con-AS, control antisense oligos; GFP, green fluorescent protein; GVBD, germinal vesicle breakdown; IP, immmunoprecipitation; MAPK, mitogen-activated protein kinase; mPR, membrane progesterone receptor; ns, not significant; P4, progesterone; Plk1, Polo-like kinase 1; PTB, phosphotyrosine-binding domain; t-AKT, total Akt; Tub, tubulin; WT, wild type.

APPL1 was first identified as an interacting protein of Akt2 via its PTB domain [34, 35], and its role in partitioning Akt2 into endosomes is well established [36–40]. Therefore, to test for the involvement of Akt2 in mPR signaling, we first knocked down endogenous Akt2 mRNA specifically without affecting Akt1 mRNA (S3C Fig). At the protein level, using an antibody that recognizes total Akt, we observe a 32% ± 3.5% decrease following Akt2 knockdown (S3D Fig). Knocking down Akt2 inhibits P4-induced oocyte maturation (Fig 3A), a phenotype that could not be rescued by overexpressing APPL1 (Fig 3A), indicating that Akt2 signals downstream of APPL1. A previous study ruled out a role for Akt1 in P4-mediated oocyte maturation but did not test for Akt2 [41]. We further confirmed biochemically the inability of oocytes to mature following Akt2 knockdown by assaying the activation of MAPK, Plk1, and Cdc2, with tubulin as the loading control (Fig 3B). Similar to APPL1, Akt2 knockdown strongly inhibited Plk1 phosphorylation and to a lesser extent, MAPK phosphorylation (Fig 3B and 3C), supporting the conclusion that Akt2 signals downstream of APPL1.

To determine whether Akt2 is recruited by APPL1 following P4 treatment, we overexpressed APPL1-GFP, pulled it down using anti-GFP beads, and quantified the levels of interacting Akt before and after P4 treatment. Akt binds to APPL1 at rest, and this interaction increases significantly but transiently following P4 treatment (Fig 3D). As with APPL1-mPR interaction, the timing to maximal interaction levels varied between different donor frogs but was always within the 2–30-minute window (Fig 3E).

To evaluate the formation of the ternary interaction between mPR, APPL1, and Akt2, we pulled down mPR and tested for Akt and APPL1 (Fig 3F, 3G and 3H). At rest, mPR interacts with APPL1 and to a lesser extent to Akt (Fig 3F). P4 treatment stimulates the formation of the mPR-APPL1-Akt complex, with peak interaction occurring between 2–30 minutes (Fig 3G). As expected for a ternary complex, the timing of maximal interaction for APPL1 and Akt invariably followed the same time course as shown in the example in Fig 3H, although this timing varied between different frogs. Importantly, the interaction between mPR and Akt was dependent on APPL1, as it was lost when APPL1 is knocked out (Fig 3I and S3B Fig), arguing that APPL1 bridges mPR-Akt2 interaction.

We then tested whether Akt recruitment to the mPR-APPL1 complex results in its activation by assessing Akt phosphorylation on serine (Ser) 473 [42]. Akt phosphorylation follows the same time course as APPL1 and Akt recruitment to the ternary complex peaking between 2–30 minutes depending on the donor female (Fig 3J and 3K), without any changes in total Akt levels (Fig 3J). Importantly, the increased Akt phosphorylation in response to P4 is lost when APPL1 is knocked down (Fig 3K), showing that APPL1 is required for Akt activation in response to P4. We further confirmed that Akt is phosphorylated at rest and in response to P4 by treating lysates with calf intestinal phosphatase (CIP) and showing that reactivity to the anti-phospho-Ser473 antibody is lost (S3E Fig). Collectively, our data position APPL1 and Akt2 downstream of mPR to release oocyte meiotic arrest.

### P4 induces mPR endocytosis in a clathrin-dependent fashion

**Fig 4:**
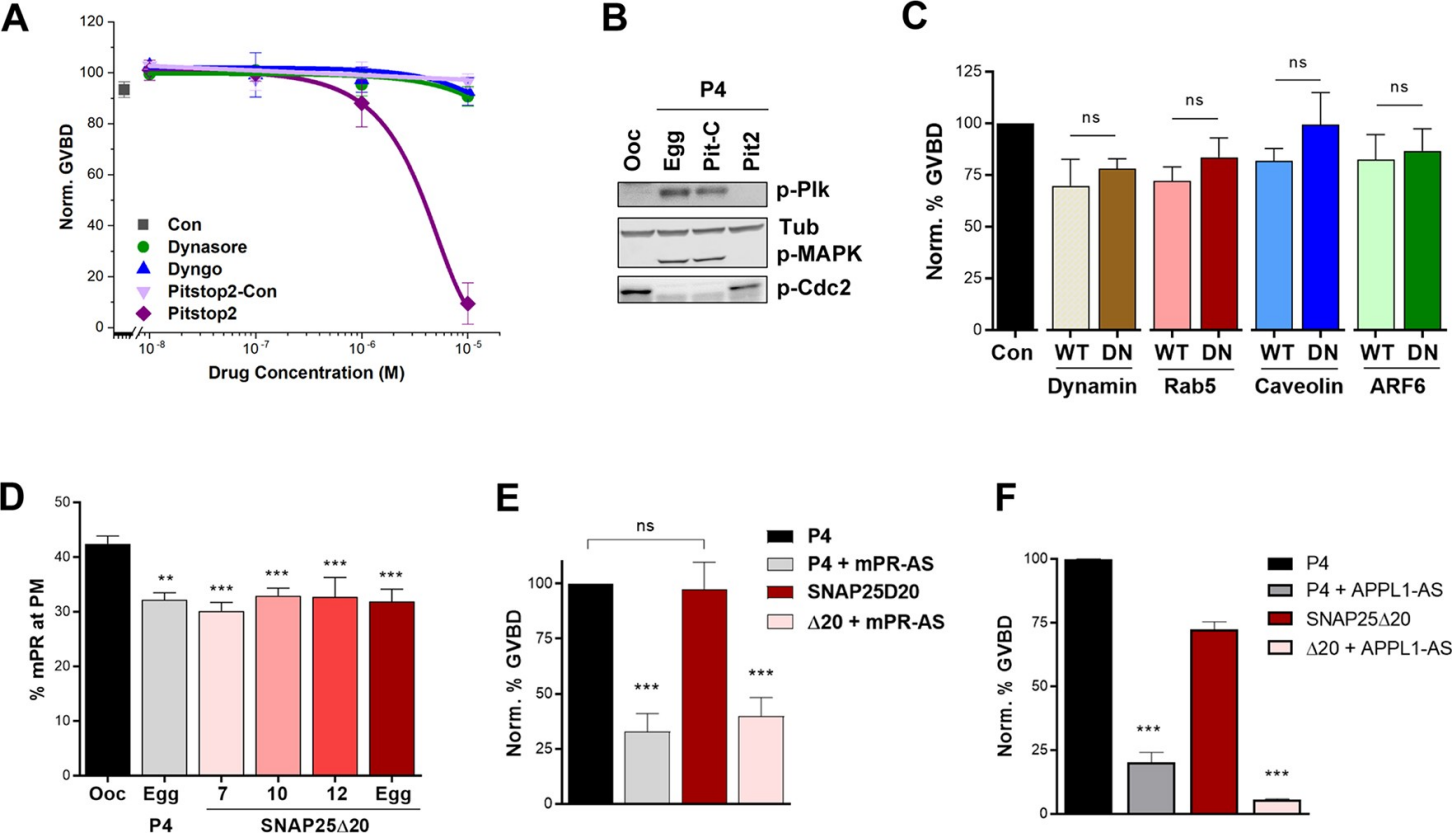
mPR is internalized through clathrin-dependent endocytosis in response to P4. (A) Oocyte maturation in response to P4 after 4 hours pre-incubation with incremental concentrations of different compounds as indicated. Con: untreated oocytes (mean ± SEM; *n =* 3–5 donor females). (B) Activation of the MAPK and Plk cascades in the different indicated conditions. Tub is shown as a loading control. (C) GVBD normalized to untreated oocytes (Con) in oocytes overexpressing WT or DN forms of dynamin, Rab5, caveolin, or ARF6 following overnight treatment with P4. For the overexpression, oocytes were injected with 20 ng RNA/oocytes for all the clones. (mean ± SEM; *n =* 3–9 donor females per condition). (D) Percent of the total mPR population at the plasma membrane (PM) in oocytes and eggs at the indicated conditions. Oocytes were injected with 40 ng SNAP25Δ20 (mean ± SEM; *n =* 7–27 oocytes per condition, from 3 donor females). (E-F) Oocyte maturation in response to P4 or SNAP25Δ20 (Δ20) (40 ng/oocyte) following knockdown of mPRβ (mPR-AS) (mean ± SEM; *n =* 4 donor females) (E) or APPL1 (APPL1-AS) (F) (mean ± SEM; *n =* 3 donor females). **p < 0.01, ***p < 0.001. Refer to S1 Data file. ARF6, ADP-ribosylation factor 6; DN, dominant-negative; Egg, P4-matured eggs; GVBD, germinal vesicle breakdown; MAPK, mitogen-activated protein kinase; mPR, membrane progesterone receptor; ns, not significant; Ooc, untreated oocytes; Pit-C, Pitstop2 control; Pit2, Pitstop2; P4, progesterone; Rab5, Ras-related protein Rab-5A; SNAP25Δ20, dominant-negative synaptosome associate protein 25; Tub, tubulin; WT, wild-type.

APPL1 regulates signaling specificity by integrating protein–protein interactions after internalization of receptors into signaling endosomes [33, 43]. Therefore, we asked whether mPR internalization is required for its signaling. We previously showed that inhibiting clathrin-mediated endocytosis using monodansylcadaverine (MDC) hinders P4-induced meiosis resumption [44]. Consistently, blocking clathrin-dependent endocytosis using Pitstop-2 (but not its control, Pitstop-control) dose-dependently blocks oocyte maturation (Fig 4A). Pitstop2 inhibits the activation of the kinase cascade downstream of P4, preventing Plk1, MAPK, and MPF activation (Fig 4B). In contrast, blocking dynamin using Dyngo has no effect on oocyte maturation (Fig 4A). Dynamin is a GTPase that is important in vesicle pinching during clathrin-dependent endocytosis [45]. We have confirmed the effectiveness of both Pitstop2 and Dyngo in terms of inhibiting endocytosis at the concentrations used in *Xenopus* oocytes using transferrin internalization (S4A–S4D Fig). In contrast to Dyngo, another dynamin inhibitor Dynasore was ineffective at inhibiting transferrin uptake in the oocyte (S4D Fig) and had no effect on GVBD (Fig 4A).

Consistent with the pharmacological inhibitors data, overexpression of the WT or dominant-negative forms of dynamin has no effect on maturation (Fig 4C). We further tested the role of Ras-related protein Rab-5A (Rab5), Caveolin, and ADP-ribosylation factor 6 (AFR6). We have previously validated the effectiveness of these dominant-negative clones in *Xenopus* oocytes [46] and further show here that the dominant-negative dynamin inhibits transferrin uptake (S4E–S4G Fig). In each case, overexpression of the respective dominant-negative mutant did not affect the ability of oocytes to undergo meiosis in response to P4 (Fig 4C), showing that endocytosis to induce maturation requires clathrin but not dynamin, caveolin, ARF6, or Rab5.

Moreover, we previously showed that blocking exocytosis, using a dominant-negative synaptosome associate protein 25 (SNAP25) mutant, releases meiotic arrest in the absence of P4 [44]. SNAP25 is an essential component of the 4-helix bundle SNAP receptor (SNARE) complex that is required for vesicle fusion during exocytosis [47]. The dominant-negative SNAP25Δ20 lacks the last 20 residues, thus removing one of the 4 helices required for vesicle fusion, and as such, blocks exocytosis when overexpressed without affecting endocytosis [44, 48, 49]. SNAP25Δ20 overexpression (S5A Fig) results in decreased membrane capacitance as a reporter of total PM area (S5B Fig), confirming the block of exocytosis but not endocytosis [44]. This induces a decrease in mPR PM residence to similar levels as in mature eggs (Fig 4D). In contrast, the Ca^2+^-activated Cl channel (TMEM16A), a typical marker for the PM that is not internalized during oocyte maturation [46], shows slight internalization in response to SNAP25Δ20 (S5C Fig). Interestingly, mPR internalization appears to be complete 7 hours after SNAP25Δ20 injection (Fig 4D) and does not increase further during maturation, hinting that the early mPR internalization in response to SNAP25Δ20 expression is enough to release meiotic arrest.

SNAP25Δ20 alone induces oocyte maturation in the absence of any P4 exposure (Fig 4E) [44]. Interestingly though, SNAP25Δ20-dependent oocyte maturation requires mPR, as it is blocked when mPR is knocked down (Fig 4E). These data indicate that the internalization of mPR is essential for its ability to induce oocyte maturation. SNAP25Δ20 fails to induce oocyte maturation when APPL1 is knocked down (Fig 4F), showing that mPR internalized following SNAP25Δ20 expression requires APPL1 to signal.

**Fig 5:**
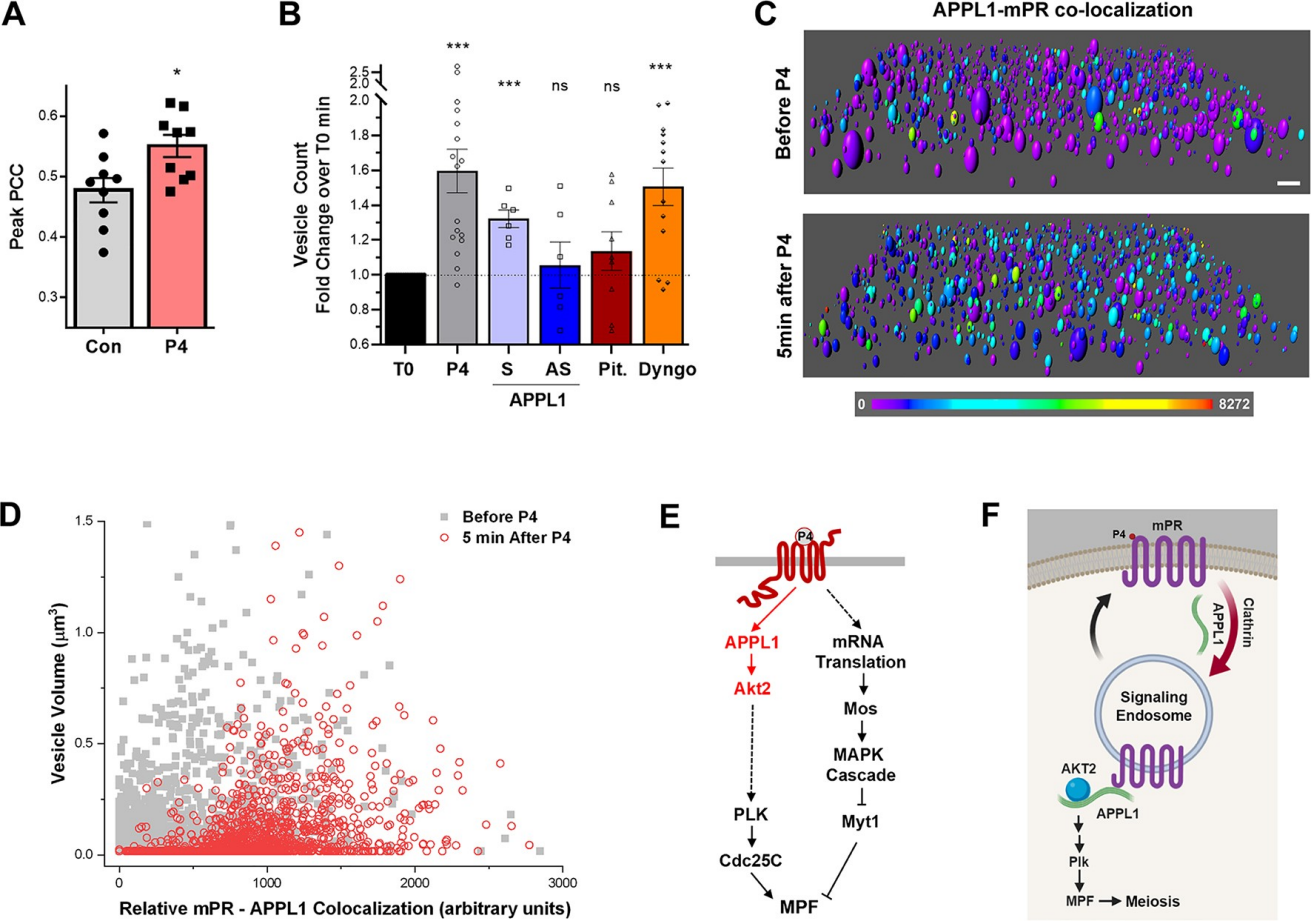
APPL1-mPR colocalization is essential to mPR internalization in clathrin-dependent–dynamin-independent way. (A) Peak PCC values measured in oocytes overexpressing mPR-GFP (20 ng/oocyte) with APPL1-mCherry (20 ng/oocyte), before (Con) and after P4 treatment (mean ± SEM; *n =* 9). (B) Fold increase in mPR-GFP-positive vesicles (oocytes were injected 20 ng of mPR-GFP RNA/oocyte) in response to P4. Each oocyte was imaged before P4 (T0) and 40 minutes after P4 and vesicles were counted across the z-stack 3D volume. Each oocyte served as its own control before P4 addition for normalization (mean ± SEM; *n* = 6–17 oocytes per condition). (C) Rendition of a confocal z-stack of images from an oocyte expressing APPL1-Ch and mPR-GFP before and 5 minutes after P4 treatment. Vesicles were rendered using Imaris software, and the heat map represents the colocalization intensity of the Cherry and GFP probes. Scale bar 2 μm. (D) Distribution of vesicle volume as a function of APPL1-mPR colocalization intensity calculated as in panel (C). (E) Signaling cascade downstream of mPR. (F) Cartoon model of P4-mPR signaling upstream of triggering the Plk1 cascade. * *p* < 0.05, *** *p* < 0.001. Refer to S1 Data file. APPL, adapter protein containing Pleckstrin homology domain, Phosphotyrosine binding domain and Leucine zipper motif 1; APPL1-S: oocytes injected with APPL1 sense oligos after P4; APPL1-AS: oocytes injected with APPL1 antisense oligos after P4; AS, antisense; control antisense oligos; Dyngo, oocytes treated with Dyngo (10^−5^ M) 4 hours prior to adding P4; GFP, green fluorescent protein; IP, immmunoprecipitation; MAPK, mitogen-activated protein; Mos, proto-oncogene serine/threonine kinase; MPF, maturation promoting factor; mPR, membrane progesterone receptor; Myt1, membrane-associated tyrosine- and threonine-specific cdc2-inhibitory kinase; ns, not significant; P4, progesterone; PCC, Pearson colocalization coefficient; Pit, oocytes treated with Pitstop (10^−5^ M) 4 hours prior to adding P4; Plk1, Polo-like kinase 1; PTB, phosphotyrosine-binding domain; t-AKT, total Akt; T0, normalized baseline; WT, wild type.

Consistent with the immunoprecipitation data, we observe increased colocalization between overexpressed mPR-GFP and APPL1-cherry after P4 exposure (S5D Fig), and this is apparent by the increased Pearson colocalization coefficient (PCC) following P4 (Fig 5A). However, this colocalization was transient and not complete, as many mPR-containing vesicles do not colocalize with APPL1 (S5D Fig). To further assess this colocalization, we rendered z-stacks of images and color-coded colocalization intensity of pixels positive for both APPL1-Ch and mPR-GFP using Imaris software (Fig 5C). This approach reveals the P4-dependent increase in APPL1-mPR colocalization in response to P4 across the entire z-stack volume (Fig 5C and 5D). These results argue for a limited and transient co-occupancy of mPR and APPL1 within signaling endosome in response to P4.

### APPL1 contributes to mPR internalization

We were intrigued by the finding that P4-dependent mPR internalization is dynamin independent but clathrin-dependent, because clathrin-dependent endocytosis typically requires dynamin. Recently, however, BAR-domain-containing proteins (such as amphiphysin) were found to be potent drivers of membrane fission and endocytosis in the absence of dynamin [50]. APPL1 contains such a BAR domain (Fig 2F), raising the possibility that it could support mPR internalization through the clathrin-dependent pathway. To test whether this is the case, we quantified the number of mPR-GFP positive vesicles as a marker for mPR endocytosis and vesicle formation (Fig 5B). Each oocyte was imaged before P4 and 40 minutes after P4, and the fold increase on per oocyte level was quantified (Fig 5B). In control oocytes (P4), oocytes injected with APPL1 sense oligos (APPL1-S), and in oocytes treated with the dynamin inhibitor Dyngo, P4 increases the number of mPR-positive vesicles, consistent with mPR internalization (Fig 5B). This increase in mPR-positive vesicles is lost, however, in oocytes injected with APPL1 antisense oligos to knock down APPL1 and in oocytes treated with the clathrin inhibitor Pitstop (Pit.) (Fig 5B). This argues that APPL1 is required for mPR internalization and mPR-positive vesicle enrichment. It further confirms the involvement of clathrin but not dynamin in mPR internalization in response to P4.

Taken together, these results show that mPR internalization through the clathrin-dependent endocytic pathway is required for its signaling. Furthermore, surprisingly, APPL1, in addition to transducing the signal to Akt2, may play a role in mPR internalization, possibly by substituting for dynamin.

## Discussion

Nongenomic P4 signaling via mPRs has emerged as an important regulator of many physiological processes, including reproductive, neuroendocrine, neurological, and immune functions [2–4]. Furthermore, mPRs have been implicated in various pathological conditions, including diabetes [51], male infertility by regulating sperm motility [52], as well as breast, ovarian, and endometrial cancers [53–55]. In addition to mPRs, other effectors have been associated with nongenomic P4 signaling, including progesterone membrane receptor component (PGMRC) [56] and alpha/beta hydrolase domain-containing protein 2 (ABHD2) [57]. mPRs exhibit broad tissue expression that often overlaps with that of nuclear PRs and bind P4 with high affinity (K_d_ 3–7 nM) [2, 6, 58]. Therefore, understanding mPR signaling is important to specifically dissect their physiological and pathological contributions.

Herein, we use frog oocyte meiosis as a prototypical physiological response requiring mPRβ to dissect the signaling cascade downstream of mPR. We show that mPRβ plays an important role in mediating the release of oocyte meiotic arrest (Fig 1F and 1G) and that P4 mediates mPR internalization through the clathrin-dependent endocytic pathway (Fig 4A, 4B and 4C). In fact, mPR internalization is sufficient for its signaling, because inducing mPR endocytosis in the absence of P4 using SNAP25Δ20 is necessary and sufficient to induce oocyte maturation (Fig 4D, 4E and 4F). Surprisingly, and although mPR endocytosis requires clathrin, it is dynamin independent (Fig 4A and 4C). Rather, APPL1 appears to substitute for dynamin, because enrichment of mPR-positive endocytic vesicles in response to P4 requires APPL1 (Fig 5B). This argues that, in addition to transducing mPR signaling, APPL1—most likely through its BAR domain—is involved in vesicle fission (Fig 5B). There is precedent for this in the literature, with BAR domains known to sense and induce membrane curvature [59], and BAR-domain-containing proteins mediating vesicle fission in the absence of dynamin [50].

Once internalized, mPR transiently interacts with APPL1 and Akt2 in signaling endosomes (Figs 2, 3, 5A, 5B, 5C and 5D). Both APPL1 and Akt2 are essential for P4-mPR signaling to induce reentry into meiosis, and they seem to preferentially induce the Plk1 arm of kinase cascades activated during oocyte maturation (Fig 2I, 2J and 2K, Fig 3B and 3C, and Fig 5E). This is consistent with Cdc25C representing the rate-limiting step in MPF activation and entry into meiosis [60]. Furthermore, the mPR-APPL1-Akt2 interaction is transient on the time scale of minutes, as shown by both immunoprecipitation experiments (Figs 2 and 3) and by imaging (Fig 5A, 5C and 5D). The ternary interaction occurs during the first 30 minutes after P4 addition (Figs 2 and 3). This timing is important as it matches the window of action necessitating P4 presence [61]. Incubating oocytes with P4 for 30 minutes is sufficient to induce maximal maturation; however, shorter incubation times result in only a subset of the population committing to meiosis [61]. Collectively, these data support a model in which mPR enriches in signaling endosomes during the first 30 minutes post P4, through clathrin-dependent endocytosis, where it interacts with APPL1 and Akt2 to induce maturation (Fig 5F). This shift is dynamic, and it appears that the cell integrates the signal from mPR-APPL1-Akt2 for minutes (5–10 minutes) before it fully commits to meiosis. Entry into meiosis is a one-way street, so oocytes need to ensure proper signaling upstream before committing, as spurious activation would lead to cell death. The dynamic endocytosis and transient enrichment of mPR in signaling endosomes over the time scale of several minutes would allow integration of the signal before committing to meiosis.

Furthermore, the proposed model focuses on the early steps downstream of mPR and does not encompass the complexity of the multiple signaling pathways that have been implicated in the release of *Xenopus* oocyte meiotic arrest in addition to mPR signaling, including G-protein coupled receptors, cAMP-PKA, and RNA polyadenylation to regulate translation to name a few (reviewed in [15, 62, 63]). In addition, to significant crosstalk between the different signaling cascades, including the Mos-MAPK and Plk1 kinase cascades [64]. Indeed, the timing of APPL1-Akt2 activation (minutes) and that of the first known downstream effector Plk1 (hours) argues for additional signaling steps and crosstalk with other pathways.

Our results are the first to show that APPL1 functions as an adaptor to mPRs. This is significant because APPL1 is well established as a signal integrator in signaling endosomes and interacts with multiple effectors (a growing list that includes at least 33 effectors known to date) [33], thus increasing the potential signaling diversity downstream of mPRs. One such effector is the small GTPase Rab5 with which APPL1 localizes in signaling endosomes [65]. Indeed, in the oocyte, APPL1 does co-localize to Rab5-positive endosomes but not exclusively, as a subset of APPL1-positive/Rab5-negative endosomes are also detected (S6A and S6B Fig). As shown in Fig 4C, Rab5 does not appear to be involved in mPR signaling; rather, mPRβ preferentially signals through Akt2, another established APPL1 effector.

APPL1 has also been shown to signal through very early endosomes (VEEs), which represent a distinct vesicular compartment from early endosome and are characterized by a smaller diameter (approximately 400 nm) and being Rab5-negative [66, 67]. We tested whether overexpressed mPR localizes preferentially to VEE in the oocyte. We define VEE as small endosomes that are APPL1-positive and Rab5-negative, which are detectable in confocal z-stacks in oocytes expressing APPL1-GFP and Rab5-RFP (S6A and S6B Fig). The distribution of APPL1-positive vesicles shows that VEE represent approximately 25% of the total vesicle population (S6C Fig), with an average volume of 0.045 μm^3^ (S6D Fig). We observe an equivalent mPR distribution independent of endosome size in the oocyte (S6E Fig), arguing against preferential signaling through VEEs.

APPL1 is a promiscuous adaptor protein that shows little preference for receptor classes. It associates with both the insulin [68] and adiponectin [31] receptors to mediate metabolic and vascular homeostasis [32, 69]. In addition, APPL1 interacts with GPCRs [40] and mediates physical and signaling compartmentalization to subsets of signaling endosomes [67]. Here we show that APPL1 interacts with mPRs to mediate their downstream signaling. The signal transduction cascade downstream of mPRs remains controversial, with significant evidence in the literature supporting a role for heterotrimeric G-proteins and yet, competing results arguing against G-protein involvement (reviewed in [3, 4, 8, 70]. The involvement of APPL1 in transducing mPR signals raises the possibility that it bridges G-protein-coupled and non-G-protein-dependent signaling at the level of signaling endosomes, potentially explaining the competing findings.

In summary, our study identifies APPL1 as an effector of mPR signaling and suggests a role for APPL1 in mPR endocytosis. It further resolves the early signaling steps downstream of mPR, a long-standing problem in understanding P4 signaling to induce oocyte meiosis. In addition, we show that mPRβ signals through signaling endosomes after its clathrin-dependent endocytosis. These results have broad implications on our understanding of progesterone biology under both physiological and pathological conditions.

## Methods

### *X*. *laevis* oocytes

Stage VI *X*. *laevis* oocytes were obtained as previously described [71]. Oocytes were maintained in L-15 medium solution (Sigma-Aldrich Cat# L4386) supplemented with HEPES (Sigma-Aldrich Cat# H4034), 0.1% (v/v) of penicillin/streptomycin stock solution (ThermoFisher Scientific Cat# 15140–122) and 0.1% (v/v) of gentamycin (EMD Millipore Cat# 345814-1GM) at pH 7.6.

### Ethics statement

All animal procedures and protocols were performed in accordance with the University of Weill Cornell Medicine Qatar Institutional Animal Care and Use Committee IACUC approved procedures (protocol #2011–0035).

### Cell culture

CHO cells were a gift from Tim McGraw (Weill Cornell Medicine) [72] and were maintained in high-glucose DMEM (ThermoFisher Cat# 11965–092) supplemented with 10% heat-inactivated FBS (ThermoFisher Cat# 10082147) and 1% (v/v) of the penicillin/streptomycin stock solution and cultured at 37°C and 5% CO_2_.

### Reagents and primers

See S1 Table for detailed list of antibodies, siRNA, select chemicals, and other critical reagents used in this study.

See S2 Table for the list of the primers used for the generation of the different clones, for real-time PCR studies, as well as the oligoes sense/antisense sequences.

For the knockdown experiments, 5–6 antisense oligos spanning the transcript of interest were tested for knockdown efficiency to select the most potent antisense for knockdown. S3 Table lists the antisense oligos tested against the different transcripts.

### Molecular biology

Coding sequences for PAQR8.L encoding the *Xenopus* mPRβ (Xenbase: XB-GENE-964793), mPR.L (NM_001085861.1), APPL1.L (NM_001090077.1) were synthetized tagged or not GFP or mCherry as indicated and cloned in pSGEM by Mutagenex Inc. pSGEM-SNAP25Δ20, pSGEM-Rab5-mCherry, pSGEM-KDEL-mCherry, pSGEM-VLDLR-mCherry, pSGEM-TMEM-mCherry, pSGEM-dynamin WT or K44A (DN), pSGEM-ARF6 WT or T22N (DN), pSGEM-Rab5 WT or S34N (DN) and pSGEM-Caveolin WT or P168L (DN) were previously described [29, 44, 46]. To generate mPR-GFP ΔN (1–70) a.a. 71–353 from mPR-GFP were PCR amplified and EcoRI/XhoI restriction sites inserted using specific primers and subcloned into pSGEM. For mPR-GFP ΔC a.a. 342–353 were deleted using the QIAGEN LongRange PCR Kit. To introduce the H129A, D146A, H281A, and H285A mutations within the zinc binding domains in pSGEM-mPR-GFP, the XL QuikChange mutagenesis kit (Agilent Technologies) was used. All constructs were verified by DNA sequencing and by analytical endonuclease restriction enzyme digestion. mRNAs for all the pSGEM clones were produced by in vitro transcription after linearizing the vectors with NheI, KpnI (pSGEM-dynamin WT), or SphI (pSGEM-dynamin DN) using the mMessage mMachine T7 or SP6 kit (Ambion). mPR-GFP and GFP-mPR were subcloned from pSGEM into pcDNA3 using the EcoRI/XhoI sites. To generate HA-XmPR or mPR-HA in pcDNA3, untagged mPR in pSGEM was PCR amplified, and BamHI-HA/XbaI or BamHI/HA-XbaI were inserted. The PCR products were then subcloned into BamH1/XbaI of pcDNA3. Relative expression of APPL1, Akt1, Akt2, and mPR were assessed by quantitative real time PCR (Affymetrix), with *Xenopus* Ornithine decarboxylase (xODC) as the internal control to normalize mRNA transcript levels [73].

### mPR N- and C-terminus topology studies in CHO cells

For transient transfection, cells were grown to 50% to 70% confluence. To determine the N- and C-terminal orientations of mPR, CHO cells plated on poly-D-lysine–coated glass-bottom plates (MatTek Corporation) were transfected with 1 μg of either pcDNA3-GFP-mPR, pcDNA3-mPR-GFP, pcDNA3-HA-mPR DNA, or pcDNA3-mPR-HA DNA using lipofectamine 2000. Forty-eight hours after transfection, cells were fixed in freshly prepared 4% PFA for 10 minutes at room temperature (RT), blocked in PBS containing 1% BSA and 0.15 M glycine for 1 hour at RT, washed in PBS, and incubated with rabbit anti-GFP antibody, diluted to 1:500 in PBS containing (2% BSA, 2% FBS) overnight at 4°C or with rabbit anti-MEK1/2. Cells were then washed with PBS and incubated with Alexa 546-conjugated anti-rabbit antibody diluted to 1:400 in PBS containing 2% BSA and 2% FBS. Cells expressing HA-tagged-mPR were either nonpermeabilized, or permeabilized for 10 minutes in PBS containing 0.1% (w/v) Triton X-100 at RT. Cells were then washed and incubated with monoclonal α-HA antibody at 1:300 diluted in PBS containing 5% FBS for 45 minutes at 37°C. Cells were washed and stained with Cy3-conjugated anti-mouse antibodies [22]. The cell membrane was stained with WGA-Alexa633 (5 μg/ml) in PBS for 15 minutes at RT. Imaging was performed on a confocal microscope (Leica) using 63× 1.4 NA oil objective. The FPP technique was done as described [20]. Briefly, CHO cells were grown on glass-bottom dishes to 50%–70% confluence before transfection with pcDNA3-GFP-mPR or pcDNA3-mPR-GFP. Forty-eight hours later, the medium was changed to NHM buffer (110 mM Na-Actetate, 20 mM HEPES, 2 mM MgCl_2_). Live imaging was performed for 1 minute followed by the addition of 0.01% of digitonin prepared in the NHM buffer. Three minutes later, 2.6 mM trypsin was added with continuous imaging at 1 frame/second. Image J software was used for analysis.

### mPR 3D modeling

Template search with BLAST (Basic Local Alignment Search Tool) and HHblits (remote homology detection method based on iterative HMM-HMM (hidden Markov model) comparison) were performed against the SWISS-MODEL template library (SMTL). The amino acid sequence of mPR sequence was obtained from SWISSPROT Sequence Data Bank. The target sequence was searched with BLAST against the primary amino acid sequence contained in the SMTL. A total of 5 templates were found. An initial HHblits profile was built using the procedure outlined in [74], followed by 1 iteration of HHblits against NR20. The obtained profile was then searched against all profiles of the SMTL. A total of 13 templates were found. For each identified template, the template's quality was predicted from features of the target-template alignment. The templates with the highest quality were then selected for model building. Then, the molecular structure of human AdipoR2 (PDB ID 5LX9) in complex with a C18 free fatty acid at 2.4 Å resolution obtained by X-ray crystal structure analysis was used to build a homology model of mPR. Models were built based on the target-template alignment using ProMod3. Coordinates, which were conserved between the target and the template, were copied from the template to the model. Insertions and deletions were remodeled using a fragment library. Side chains were then rebuilt. Finally, the geometry of the resulting model was regularized by using a force field. In case loop modeling with ProMod3 fails, an alternative model was built with PROMOD-II [75]. The global and per-residue model quality was assessed using the QMEAN scoring function [76]. For improved performance, weights of the individual QMEAN terms were trained specifically for SWISS-MODEL. The quaternary structure annotation of the template was used to model the target sequence in its oligomeric form. The method [77] was based on a supervised machine learning algorithm, Support Vector Machines (SVM), which combines interface conservation, structural clustering, and other template features to provide a quaternary structure quality estimate (QSQE). The QSQE score is a number between 0 and 1, reflecting the expected accuracy of the interchain contacts for a model built based on a given alignment and template. Higher numbers indicate higher reliability. This complements the GMQE score, which estimates the accuracy of the tertiary structure of the resulting model.

### Oocyte maturation and protein expression studies

The oocytes were used 24 to 72 hours after harvesting and injected with RNAs (20 to 50 ng/oocyte) or sense/antisense oligos (100 ng/oocyte) and kept at 18°C for 1–2 days to allow for protein expression or mRNA degradation. After treatment with progesterone or the synthetic P4-derivative, both at 10^−5^ M, GVBD was detected visually by the appearance of a white spot at the animal pole. For the staining with WGA, oocytes that are completely denuded from follicular cells were selected by negative staining with Hoechst 33342 and were used for the intended experiments. For the dynamin and clathrin inhibition studies, oocytes were preincubated for 4 hours with different concentration of the selective cell-permeable clathrin inhibitor Pitstop 2, Pitstop 2-negative control, Dyngo, and Dynasore, followed by overnight incubation with P4 at 10^−5^ M concentration. For all GVBD experiments, 25 to 30 oocytes were used per condition and per frog, and each experiment was repeated on multiple donor females typically 3–4 females for each experiment.

### Co-Immunoprecipitation

Around 70 oocytes injected with GFP-tagged proteins mRNA (20 ng/oocyte) were lysed in IP solution (30 mM HEPES, 100 mM NaCl [pH 7.5]) containing protease and phosphatase inhibitors (5 μl/ oocyte). Lysates were cleared of yolk by centrifugation at 1,000*g* 3 times for 10 minutes each at 4°C. Supernatants were then solubilized with 4% NP40 for 1 hour followed by 15 minutes centrifugation at 18,188 *g* at 4°C before immunoprecipitation using anti-GFP microbeads (1 μl/oocyte) per the manufacturer’s instructions. Each independent IP experiment was done using oocytes from a separate donor female frog.

### SDS-page and western blotting

For the GVBD experiments, 20 to 30 oocytes were collected from all conditions when the control condition reached maximal GVBD in response to P4. Cells were lysed in MPF lysis buffer (0.08 M β-glycerophosphate, 20 mM HEPES [pH 7.5], 15 mM MgCl_2_, 20 mM EGTA, 1 mM Na-Vanadate, 50mM NaF, 1mM DTT), in the presence of protease and phosphatase inhibitors, followed by centrifugation 3 times at 1,000 *g* for 10 minutes at 4°C to remove yolk granules. For the treatment with the CIP, 20 oocytes were lysed in IP solution (30 mM HEPES, 100 mM NaCl [pH 7.5]) (5 μl/ oocyte) containing protease and phosphatase inhibitors, and centrifuged twice at 1,000 *g* for 10 minutes each at 4°C, followed by incubating the supernatants with CIP (10 units) for 60 minutes at 37°C. For the signaling studies of MAPK/Plk1/cdc2/Akt activation, the knockdown of APPL1 and t-AKT, and the overexpression of mPR-GFP and SNAP25Δ20, lysates from an average of 5 oocytes were separated on 4%–12% or 10% SDS-PAGE gels. For IP samples, an average of 15 oocytes were loaded per condition. Proteins were transferred from SDA-PAGE gels to polyvinylidene difluoride (PVDF) membranes (Millipore), blocked for one hour at RT with 5% Milk in TBS-T buffer (150 mM NaCl and 20 mM Tris; [pH 7.6], 0.1% Tween) and then incubated overnight at 4°C in 3% BSA in TBS-T with the primary antibody. Blots were then washed 3 times with TBS-T and probed for 1 hour with horseradish peroxidase (HRP)-conjugated secondary antibody 1/10,000 (for p-Cdc2, p-Plk1, and p-AKT), or with infrared fluorescence, IRDye 800 and 680 secondary antibodies (1/10,000) (for GFP, APPL1, p-MAPK, t-AKT, SNAP25, and Tubulin). The blots were visualized using ECL-based detection of HRP followed by Image J analysis or using the LI-COR Odyssey Clx Infrared Imaging system and analyzed using LI-COR image Studio Lite v.4.0. The primary antibodies used are anti-APPL1 (1/1,000) anti-GFP (1/1,000), anti-SNAP25 (1/1,000), anti-Tubulin (1/10,000), anti-p-Plk1 (1/1,000), anti-phospho-MAPK (1/5,000), anti-phospho-Cdc2 (1/1,000), anti-t-AKT (1/1,000), and anti-p-AKTS473 (1/1,000).

### Oocytes imaging and analysis

Oocytes were imaged on a LSM880 confocal (Zeiss, Germany) fitted with a Plan Apo 63x/1.4 oil immersion objective, with z-stacks taking in 0.5-μm sections using a 1 Airy unit pinhole aperture. Images were analyzed using ZEN 2.3 (Zeiss) or the ImageJ software. To measure the distribution of mPR and TMEM at the cell membrane, WGA was used as membrane markers. For each oocyte, the percentage (%) of membrane mPR and TMEM was calculated by analyzing the intensity of fluorescence distribution through a z-stack of images, where we conservatively used 2 z-stacks below the peak of WGA fluorescence, as a reference to mark the end of the PM compartment. Data were normalized to control conditions. For the transferrin imaging experiment oocytes were incubated for 1 hour with transferring tagged with Alexa 633 in Ringer at 0.125 mg/ml (Pitstop exp) or 0.25 mg/ml (Dyngo and dynamin exp). Cells were washed before imaging. For mPR-positive vesicle count, z-stacks of images from oocytes overexpressing mPR-GFP were collected before and after P4 in the presence or absence of APPL1 antisense. ImageJ software was used to quantify mPR vesicles using the 3D objects counter application. To follow mPR-APPL1 colocalization and signaling endosomes before and after P4 treatment, image z-stacks from oocytes overexpressing mPR-GFP and APPL1-mCherry were taken before and at different time points after P4 treatment using the super resolution AIRYSCAN mode on the LSM880 (Zeiss, Germany). The Imaris image analysis software was used for the generation of APPL1 3D spots as a function of mPR colocalization and to analyze the PCC values at each z-stack and time points. Colocalization intensity was calculated by the Imaris software for each vesicle within the rendered volume as $f=\sqrt{i1*i2}$ where i1 and i2 are the intensities in the individual channels.

### Electrophysiology

Membrane capacitance was monitored using the build in routine from pClamp to measure the cell membrane area. The cells were continuously superfused with Ringer buffer (in mM: 96 NaCl, 2.5 KCl, 1.8 CaCl_2_, 2 MgCl_2_, 10 HEPES [pH 7.4]) during voltage-clamp experiments using a peristaltic pump.

### Statistics

Values are given as means ± SEM. Statistical analysis was performed when required using student paired, unpaired *t*-test or ANOVA test. *p*-values are indicated as follows: *p < 0.05, ** *p* < 0.01, ***p < 0.001, and not significant (ns). Unless indicated, each experiment was repeated at least 3 times from 3 independent donor female frogs.

## Supporting information

S1 Fig *Xenopus* mPR protein sequence alignment with human adiponectin receptor 1 and 2, its projected topology and 3D structure, and mPR knockdown effectiveness in oocytes. (A) Alignment of *Xenopus* mPRβ amino acids sequence with human and *Xenopus* AdipoRs. (B) Orthogonal extracellular view of 3D model of mPRβ based on the solved crystal structure of AdipoRs, showing its predicted 7 transmembrane domains and its predicted zinc coordination domain (Zn). (C) Superimposed 3D structures of *Xenopus* mPRβ (red) and the human AdipoR2 (blue) showing significant alignment between the 2 receptors. (D) Alignment of mPRβ.L and mPRβ.S amino acids sequences. The regions deleted in the mPR-ΔN and mPR-ΔC mutants are marked by a blue line on top of the sequence. The 7 transmembrane domains are marked by the red boxes, and the conserved putative Zn^2+^ coordinating residues that were mutated to Ala in the Zn mutant are underlined. The putative Zn coordinating residues were identified based on sequence conservation with human AdipR1 and AdipoR2. (E) mPR knockdown experiments. Oocytes were injected with Con-AS or specific mPRβ antisense (mPR-AS) oligonucleotides and incubated at 18 ^o^C for 24 hours. RNAs were prepared from 20 oocytes and analyzed by RT-PCR to determine the efficacy of mPRβ knockdown as compared to naive oocytes (Con). Data are expressed as relative RNAs levels of mPR mRNA transcripts after normalizing to xODC mRNA levels as a house keeping gene. (mean ± SEM; *n =* 3 donor females). (F) A representative western blot from naive untreated oocytes, oocytes injected with Con-AS oligos and oocytes overexpressing mPR-GFP alone (Con) or co-injected with Con-AS or mPR-AS oligos. Tubulin is used as a loading control. ***p < 0.001. Refer to S1 Data file. AdipoR, adiponectin receptor; Con-AS, control antisense; GFP, green fluorescent protein; mPR, membrane progesterone receptor; mPR-AS, mPRβ antisense; ns; not significant; RT-PCR, real-time polymerase chain reaction; xODC, *Xenopus* Ornithine decarboxylase.
(TIF)

S2 Fig Plasma membrane localization of mPR-GFP wt, ΔN, ΔC, or Zinc mutant, their colocalization with VLDLR, and sequence alignment between xenopus and human APPL1. (A) Representative images from a confocal z-stack taken across the PM plane of oocytes at different z location either at the PM plane (PM) or deep into the oocyte to visualize the ER plane. Oocytes were injected with either the full-length wild-type mPR-GFP or with the different mPR mutants: ΔN, ΔC, or Zinc mutant (Zn) (20 ng/oocyte). Oocytes were also injected with the ER marker KDEL-mCherry to visualize the ER and stained with WGA to mark the PM (20 ng/oocyte). Scale bar 2 μm. (B) Confocal images from oocytes overexpressing mPR-GFP WT, ΔN or ΔC along with VLDLR-mCherry to visualize colocalization and interaction between the different mPR mutants and the mPR trafficking chaperone VLDLR. Scale bar 2 μm. (C) Sequence alignment between *Xenopus* APPL1.S, APPL1.L, and hAPPL1. APPL1, Adapter protein containing Pleckstrin homology domain, Phosphotyrosine binding domain and Leucine zipper motif 1; ER, endoplasmic reticulum; GFP, green fluorescent protein; hAPPL1, human APPL1; mPR, membrane progesterone receptor; PM, plasma membrane; VLDLR, very-low-density lipoprotein receptor; WGA, wheat germ agglutinin.
(TIF)

S3 Fig The effectiveness of APPL1 and Akt2 knockdown on the levels of RNA (APPL1 and Akt2) and proteins (Akt2), as well as the binding of APPL1 to mPR-GFP, and validation of Akt phosphorylation at rest. (A) APPL1 antisense knockdown experiments. Oocytes were injected with control APPL1 sense or antisense oligonucleotides and incubated at 18 ^o^C for 24 hours. RNAs were prepared and analyzed by RT-PCR to determine the efficacy of APPL1 mRNA knockdown between control uninjected (Con), sense oligos (APPL1 sense) or antisense oligos (APPL1 antisense) injected oocytes. mPR mRNA levels were measured as well to rule out any effect of the APPL1 antisense oligos on mPR expression, which would affect oocyte maturation. Data are expressed as relative APPL1.L, APPL1.S, and mPR mRNA transcripts normalized to xODC mRNA levels (mean ± SEM; *n =* 3 donor females). (B) Representative western blot of mPR-GFP and APPL1 co-IP before and after APPL1 knockdown. Oocytes were left untreated (naive) or injected with mPR-GFP RNA with or without APPL1 antisense oligos (APPL1-AS). Lysates were immunoprecipitated using beads coupled to anti-GFP antibodies. Eluates from the IPs were analyzed by western blotting using APPL1 and GFP antibodies. (C) Akt2 knockdown experiments. Oocytes were untreated (naive), injected with Con-AS oligos or injected with 2 different specific Akt2 antisense oligos (AKT2-AS1 and AKT2-AS2) and incubated at 18 ^o^C for 48 hours, followed by RNA preparation and gene expression analysis by real-time PCR. Both Akt2 antisense oligos specifically degrade Akt2 mRNA without affecting the levels of Akt1 mRNA. The mRNAs levels of AKT2 and AKT1 were normalized to xODC mRNA levels as a house keeping gene (mean ± SEM; *n =* 3–7 donor females). (D) Representative western blot (right panel) and t-AKT proteins levels quantification (mean ± SEM; *n =* 7 donor females) (left panel), between control uninjected and AKT2-AS1 injected oocytes. Tubulin is used as a loading control. APPL1/Tubulin proteins ratios are normalized to the control oocytes. (E) Western blot of t-AKT, p-AKT and tubulin before (Con) or after P4 treatment, in the presence or absence of CIP. ***p < 0.001. Refer to S1 Data file. APPL1, Adapter protein containing Pleckstrin homology domain, Phosphotyrosine binding domain and Leucine zipper motif 1; CIP, calf intestinal phosphatase; Con-AS, control antisense; GFP, green fluorescent protein; hAPPL1, human APPL1; IP, immunoprecipitation; mPR, membrane progesterone receptor; ns, not significant; P4, progesterone; PM, plasma membrane; t-AKT, total AKT; VLDLR, very-low-density lipoprotein receptor; xODC, *Xenopus* Ornithine decarboxylase.
(TIF)

S4 Fig The effectiveness of Pitstop, Dyngo, dynasore, and dynamin in blocking transferrin endocytosis. (A, C, E) Representative focal plane images of oocytes incubated with Alexa-633-labeled transferrin to assess its internalization and treated overnight with Pitstop or its control (A), Dyngo or vehicle (C), or injected with either WT or DN Dynamin RNA (E). (B, D, F). Quantification of intracellular transferrin fluorescence intensity normalized to the control treatment as indicated for the different conditions. Vehicle control (Veh.), Pitstop and its control (10^−5^ M) (mean ± SEM; 15 oocytes per condition, from 3 donor females), Dyngo 10^−5^ M (mean ± SEM; 25–30 oocytes per condition, from 2 donor females), and Dynasore 10^−5^ and 3×10^−5^M (mean ± SEM; 25–29 oocytes per condition, from 2 donor females). **p < 0.01, ***p < 0.001. Refer to S1 Data file. DN, dominant-negative; WT, wild type.
(TIF)

S5 Fig Plasma membrane localization of mPR after SNAP25Δ20 expression and up to GVBD, and Colocalization of mPR with APPL1 after P4 treatment. (A) Representative western blot showing SNAP25Δ20 expression at time 0, 7, and 10 hours after RNA injection into the oocytes. Tubulin was used as the loading control. (B) Membrane capacitance of oocytes before and 7 to 10 hours after SNAP25Δ20 RNA (Δ20) injection (mean ± SEM; *n =* 7–11 oocytes per condition). (C) Quantification of the PM level of TMEM-mCherry before or after expression of SNAP25Δ20 or P4 treatment as indicated. Oocytes are cells that have not been exposed to P4, whereas eggs are oocytes treated with P4 at 2 hours after GVBD. Oocyte maturation does not affect the levels of TMEM at the PM, whereas SNAP25Δ20 expression results in a small decrease in TMEM at the PM. This decrease is relatively minor compared to the decrease in mPR at the PM in response to SNAP25Δ20 expression (Fig 4D) (mean + SEM; *n =* 7–17 oocytes per condition, from 2 donor females). (D) Representative confocal images of an oocyte overexpressing mPR-GFP (20 ng/oocyte) and APPL1-mCherry (20 ng/oocyte) before and 10 minutes after P4 treatment. The arrows indicate co-localized mPR and APPL1 vesicles. Scale bar 2 μm. *p < 0.05, ***p < 0.001. Refer to S1 Data file. APPL1, Adapter protein containing Pleckstrin homology domain, Phosphotyrosine binding domain and Leucine zipper motif 1; GFP, green fluorescent protein; GVBD, germinal vesicle breakdown; IP, immunoprecipitation; mPR, membrane progesterone receptor; P4, progesterone; PM, plasma membrane; SNAP25Δ20, dominant-negative synaptosome associate protein 25.
(TIF)

S6 FigThe study of APPL1 localization with Rab5 to define the subset of APPL1 very early endosomes, and quantification of APPL1 very early and early endosomes, before and after P4 treatment.(A) Example confocal images from an oocyte expressing APPL1-GFP (20 ng/oocyte) and Rab5-RFP (20 ng/oocyte) showing their partial colocalization. Arrows indicate smaller vesicles that are APPL1-positive but Rab5-negative. Scale bar 1 μm. (B) Rendition in 3D of a z-stack of confocal images showing the distribution of APPL1-positive vesicles across the entire stack volume. Vesicles are color-coded with the heat map indicating the colocalization intensity of individual vesicle of both APPL1-GFP and Rab5-RFP. (C) Distribution of APPL1-positive vesicles as a function of vesicle volume and colocalization with Rab5. Colocalization intensity was calculated by the Imaris software for each vesicle within the rendered volume as $f=\sqrt{i1*i2}$ where i1 and i2 are the intensities in the individual channels. Colocalization intensity was normalized to maximal colocalization. We define Rab5-negative vesicles as vesicles where the normalized colocalization in below 0.15, that is 15% of the maximal colocalization. (D) We define VEEs as APPL1-positive and Rab5-negative vesicles, which have an average volume of 0.045 μm^3^. (E) Normalized colocalization of APPL1 and mPR in VEE endosome (vesicle ≤ 0.045 μm^3^), EE (vesicles >0.045 μm^3^) and in the entire vesicle population (All) at time 0 minutes (T0) and at the time point between 2 and 30 minutes when maximal colocalization is observed in different batches of oocytes (Max). The data are normalized to T0 for each vesicle population (mean ± SEM, *n* = 7). *p < 0.05. Refer to S1 Data file. APPL1, Adapter protein containing Pleckstrin homology domain, Phosphotyrosine binding domain and Leucine zipper motif 1; GFP, green fluorescent protein; ns, not significant; VEE, very early endosome; Rab5, Ras-related protein Rab-5A.(TIF)

S1 Table List of antibodies, chemicals and reagents used in the study. (DOCX)

S2 Table List of antisense oligonucleotides and primers used in the study. (DOCX)

S3 Table List of mPR/APPL1/Akt2 antisense oligonucleotides tested in the study. The most effective antisense oligos that were used for the knockdown experiments are bolded. Akt2, protein kinase B; APPL1, Adapter protein containing Pleckstrin homology domain, Phosphotyrosine binding domain and Leucine zipper motif 1; mPR, membrane progesterone receptor.
(DOCX)

S1 Data Excel file that includes all individual numerical data in the different figure panels. (XLSX)

S1 raw images Full gel images of all western blots in the paper. (PDF)

## Abbreviations

ABHD2: alpha/beta hydrolase domain-containing protein 2
AdipoQ: adiponectin
AdipoR: adiponectin receptor
APPL1: Adapter protein containing Pleckstrin homology domain, Phosphotyrosine binding domain and Leucine zipper motif 1
ARF6: ADP-ribosylation factor 6
BSA: Bovine Serum Albumin
CHO: Chinese Hamster Ovary
CIP: calf intestinal phosphatase
CPEB: cytoplasmic polyadenylation element binding protein
ER: endoplasmic reticulum
GFP: green fluorescent protein
GVBD: germinal vesicle breakdown
M: plasma membrane
MAPK: mitogen-activated protein kinase
MDC: monodansylcadaverine
MEK: mitogen-activated protein kinase kinase
MPF: maturation promoting factor
mPR: membrane progesterone receptor
mPR-KD: mPR knockdown
P4: progesterone
PAQR: progesterone and adiponectin receptor
PGMRC: progesterone membrane receptor component
Plk1: Polo-like kinase 1, cyclin-dependent kinase 1, Cdk1
PR: P4 receptor
Rab5: Ras-related protein 5A
SNAP25: synaptosome associate protein 25
SNARE: SNAP Receptor
VEE: very early endosome
VLDLR: very-low-density lipoprotein receptor
WGA: wheat germ agglutinin
WT: wild type
