# Correction: Membrane progesterone receptor induces meiosis in *Xenopus* oocytes through endocytosis into signaling endosomes and interaction with APPL1 and Akt2

**DOI:** 10.1371/journal.pbio.3001117

**Published:** 2021-02-10

**Authors:** Nancy Nader, Maya Dib, Rawad Hodeify, Raphael Courjaret, Asha Elmi, Ayat S. Hammad, Raja Dey, Xin-Yun Huang, Khaled Machaca

The following information is missing from the Funding statement: Open Access funding was provided by the Qatar National Library.
