# Supplementary figures and images for: Membrane progesterone receptor induces meiosis in Xenopus oocytes through endocytosis into signaling endosomes and interaction with APPL1 and Akt2
Source: PLoS Biol. 2020 Nov 2;18(11):e3000901. doi: 10.1371/journal.pbio.3000901 (PMC7660923; doi:10.1371/journal.pbio.3000901)

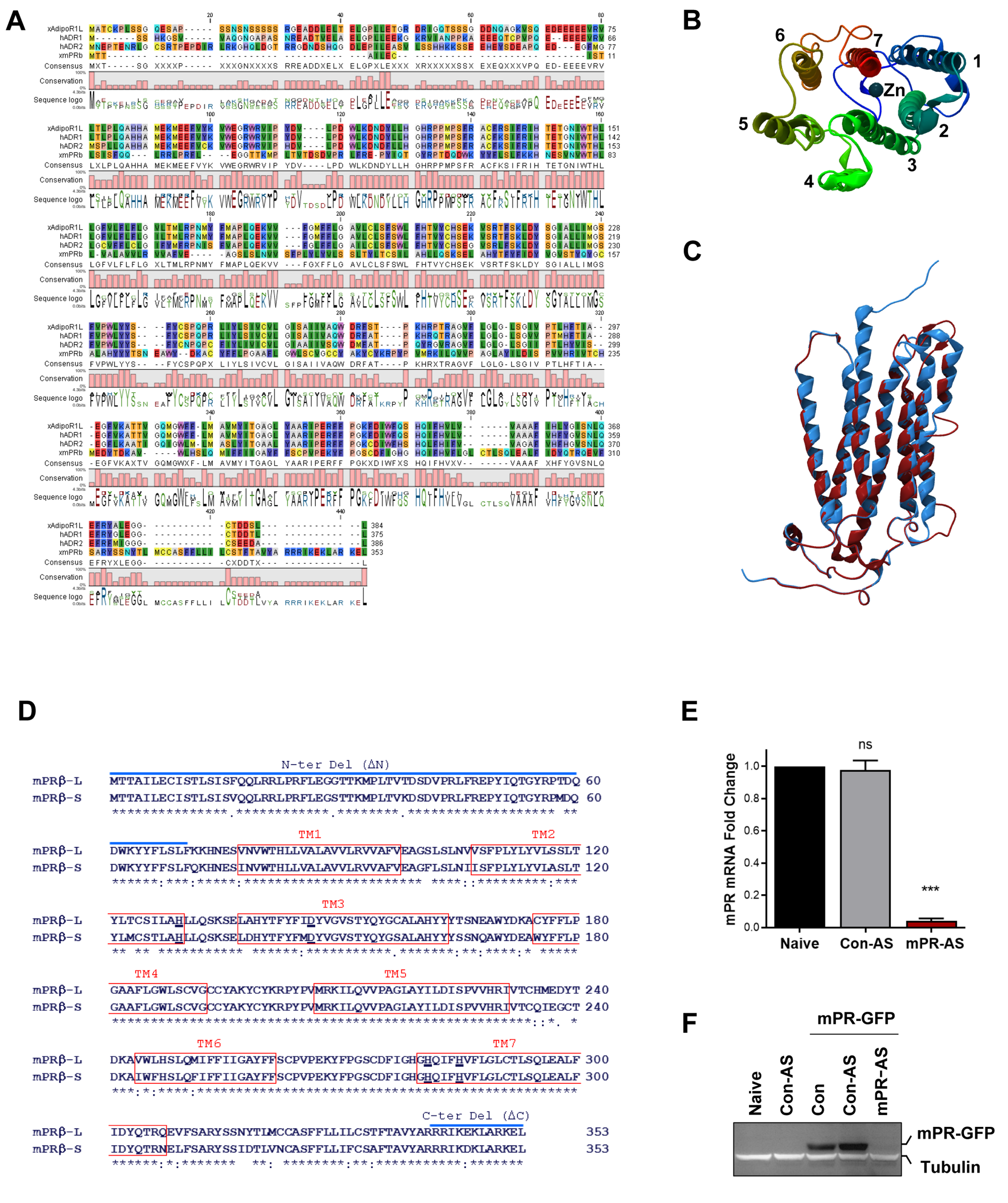

Supplement: S1 Fig — (A) Alignment of Xenopus mPRβ amino acids sequence with human and Xenopus AdipoRs. (B) Orthogonal extracellular view of 3D model of mPRβ based on the solved crystal structure of AdipoRs, showing its predicted 7 transmembrane domains and its predicted zinc coordination domain (Zn). (C) Superimposed 3D structures of Xenopus mPRβ (red) and the human AdipoR2 (blue) showing significant alignment between the 2 receptors. (D) Alignment of mPRβ.L and mPRβ.S amino acids sequences. The regions deleted in the mPR-ΔN and mPR-ΔC mutants are marked by a blue line on top of the sequence. The 7 transmembrane domains are marked by the red boxes, and the conserved putative Zn2+ coordinating residues that were mutated to Ala in the Zn mutant are underlined. The putative Zn coordinating residues were identified based on sequence conservation with human AdipR1 and AdipoR2. (E) mPR knockdown experiments. Oocytes were injected with Con-AS or specific mPRβ antisense (mPR-AS) oligonucleotides and incubated at 18 oC for 24 hours. RNAs were prepared from 20 oocytes and analyzed by RT-PCR to determine the efficacy of mPRβ knockdown as compared to naive oocytes (Con). Data are expressed as relative RNAs levels of mPR mRNA transcripts after normalizing to xODC mRNA levels as a house keeping gene. (mean ± SEM; n = 3 donor females). (F) A representative western blot from naive untreated oocytes, oocytes injected with Con-AS oligos and oocytes overexpressing mPR-GFP alone (Con) or co-injected with Con-AS or mPR-AS oligos. Tubulin is used as a loading control. ***p < 0.001. Refer to S1 Data file. AdipoR, adiponectin receptor; Con-AS, control antisense; GFP, green fluorescent protein; mPR, membrane progesterone receptor; mPR-AS, mPRβ antisense; ns; not significant; RT-PCR, real-time polymerase chain reaction; xODC, Xenopus Ornithine decarboxylase. (TIF) [file pbio.3000901.s001.tif]

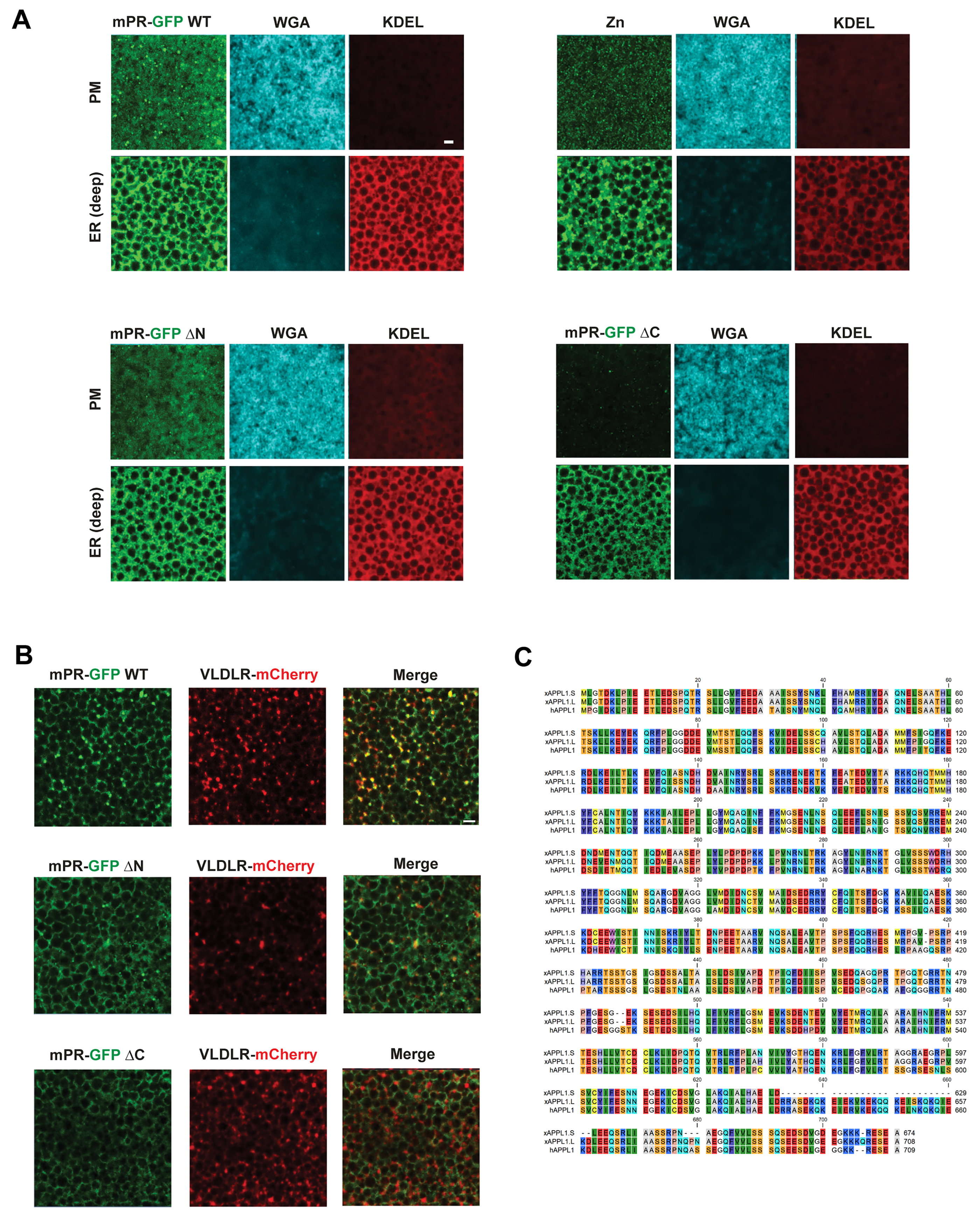

Supplement: S2 Fig — (A) Representative images from a confocal z-stack taken across the PM plane of oocytes at different z location either at the PM plane (PM) or deep into the oocyte to visualize the ER plane. Oocytes were injected with either the full-length wild-type mPR-GFP or with the different mPR mutants: ΔN, ΔC, or Zinc mutant (Zn) (20 ng/oocyte). Oocytes were also injected with the ER marker KDEL-mCherry to visualize the ER and stained with WGA to mark the PM (20 ng/oocyte). Scale bar 2 μm. (B) Confocal images from oocytes overexpressing mPR-GFP WT, ΔN or ΔC along with VLDLR-mCherry to visualize colocalization and interaction between the different mPR mutants and the mPR trafficking chaperone VLDLR. Scale bar 2 μm. (C) Sequence alignment between Xenopus APPL1.S, APPL1.L, and hAPPL1. APPL1, Adapter protein containing Pleckstrin homology domain, Phosphotyrosine binding domain and Leucine zipper motif 1; ER, endoplasmic reticulum; GFP, green fluorescent protein; hAPPL1, human APPL1; mPR, membrane progesterone receptor; PM, plasma membrane; VLDLR, very-low-density lipoprotein receptor; WGA, wheat germ agglutinin. (TIF) [file pbio.3000901.s002.tif]

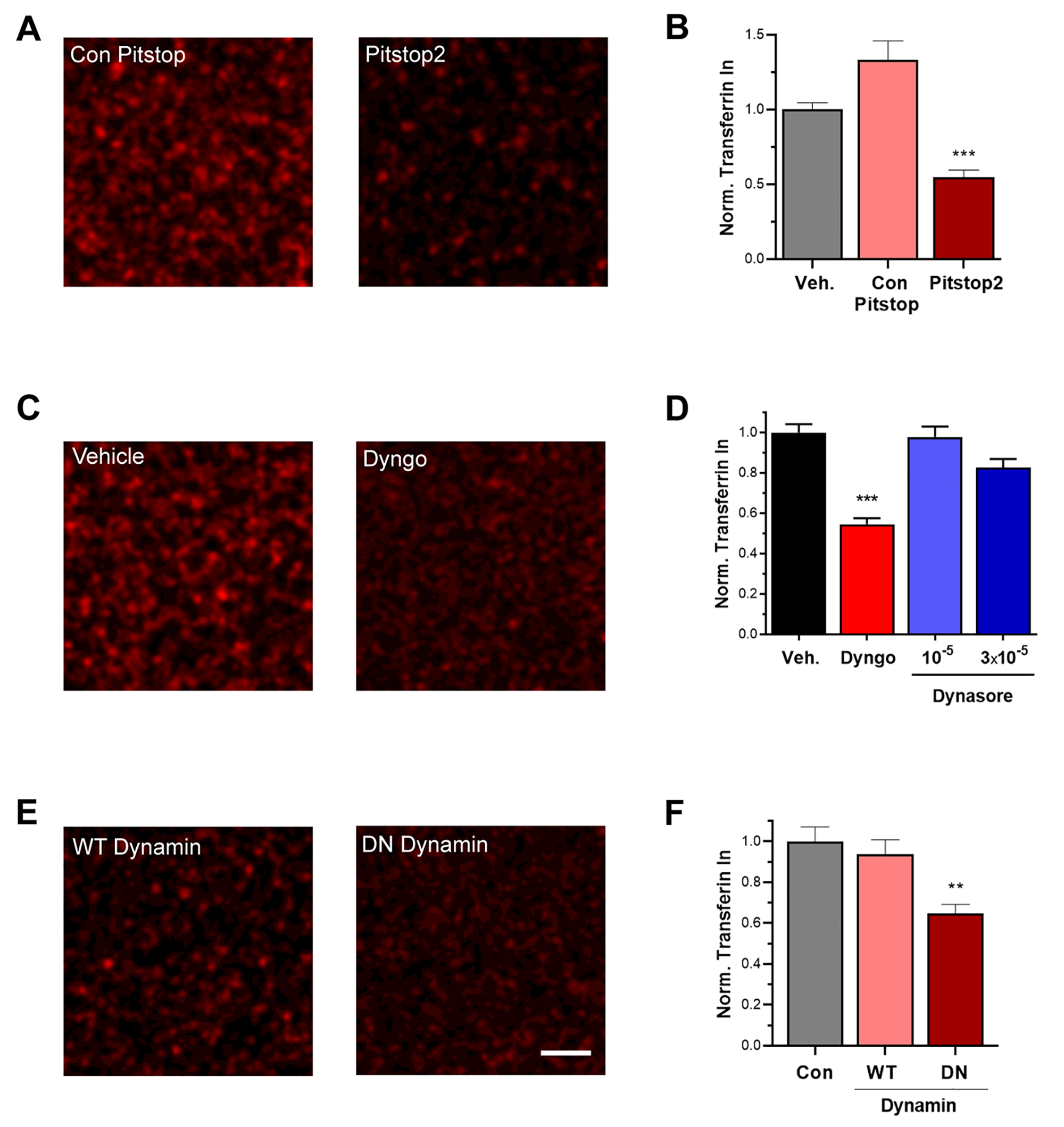

Supplement: S4 Fig — (A, C, E) Representative focal plane images of oocytes incubated with Alexa-633-labeled transferrin to assess its internalization and treated overnight with Pitstop or its control (A), Dyngo or vehicle (C), or injected with either WT or DN Dynamin RNA (E). (B, D, F). Quantification of intracellular transferrin fluorescence intensity normalized to the control treatment as indicated for the different conditions. Vehicle control (Veh.), Pitstop and its control (10−5 M) (mean ± SEM; 15 oocytes per condition, from 3 donor females), Dyngo 10−5 M (mean ± SEM; 25–30 oocytes per condition, from 2 donor females), and Dynasore 10−5 and 3×10^−5^M (mean ± SEM; 25–29 oocytes per condition, from 2 donor females). **p < 0.01, ***p < 0.001. Refer to S1 Data file. DN, dominant-negative; WT, wild type. (TIF) [file pbio.3000901.s004.tif]

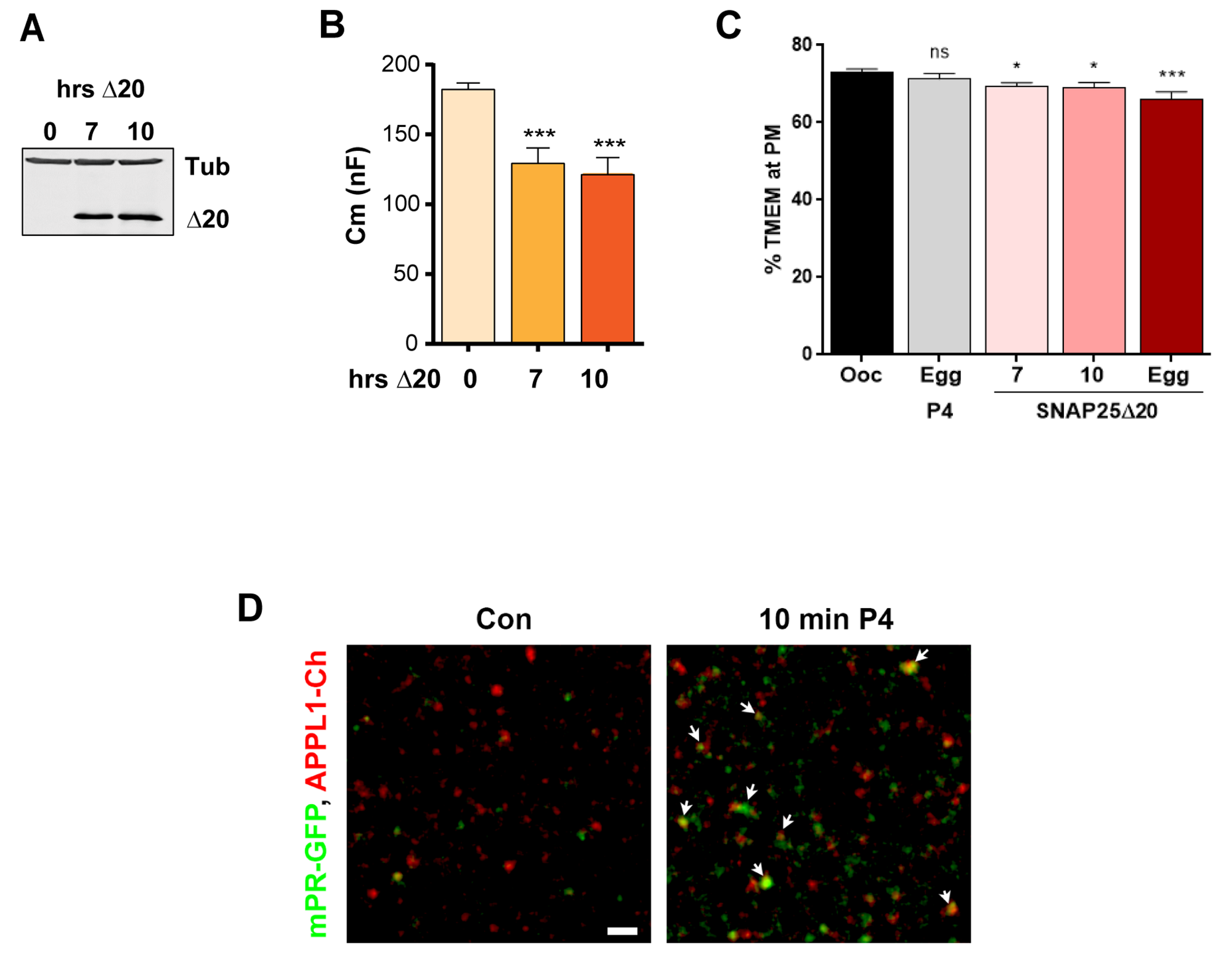

Supplement: S5 Fig — (A) Representative western blot showing SNAP25Δ20 expression at time 0, 7, and 10 hours after RNA injection into the oocytes. Tubulin was used as the loading control. (B) Membrane capacitance of oocytes before and 7 to 10 hours after SNAP25Δ20 RNA (Δ20) injection (mean ± SEM; n = 7–11 oocytes per condition). (C) Quantification of the PM level of TMEM-mCherry before or after expression of SNAP25Δ20 or P4 treatment as indicated. Oocytes are cells that have not been exposed to P4, whereas eggs are oocytes treated with P4 at 2 hours after GVBD. Oocyte maturation does not affect the levels of TMEM at the PM, whereas SNAP25Δ20 expression results in a small decrease in TMEM at the PM. This decrease is relatively minor compared to the decrease in mPR at the PM in response to SNAP25Δ20 expression (Fig 4D) (mean + SEM; n = 7–17 oocytes per condition, from 2 donor females). (D) Representative confocal images of an oocyte overexpressing mPR-GFP (20 ng/oocyte) and APPL1-mCherry (20 ng/oocyte) before and 10 minutes after P4 treatment. The arrows indicate co-localized mPR and APPL1 vesicles. Scale bar 2 μm. *p < 0.05, ***p < 0.001. Refer to S1 Data file. APPL1, Adapter protein containing Pleckstrin homology domain, Phosphotyrosine binding domain and Leucine zipper motif 1; GFP, green fluorescent protein; GVBD, germinal vesicle breakdown; IP, immunoprecipitation; mPR, membrane progesterone receptor; P4, progesterone; PM, plasma membrane; SNAP25Δ20, dominant-negative synaptosome associate protein 25. (TIF) [file pbio.3000901.s005.tif]

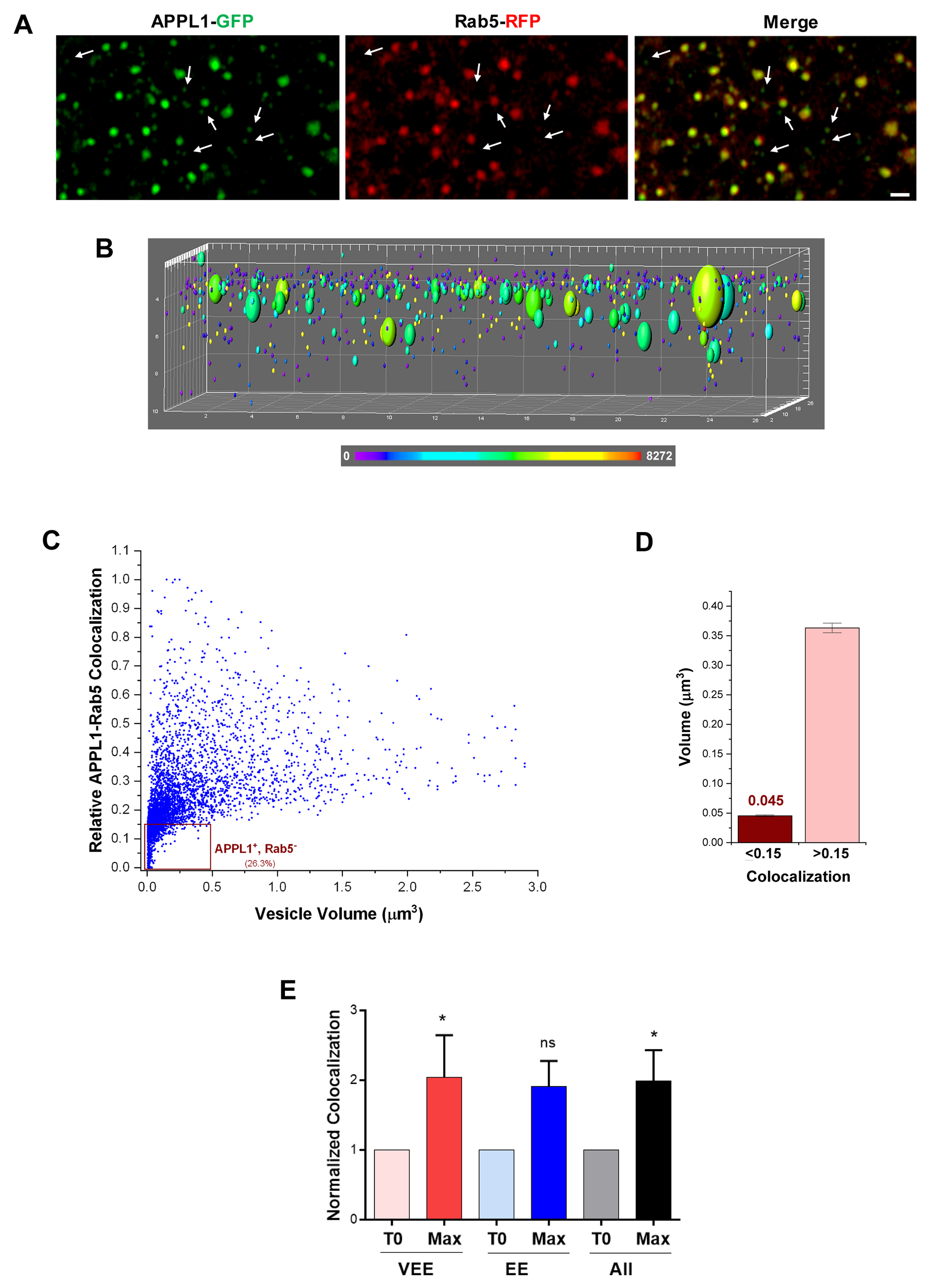

Supplement: S6 Fig — (A) Example confocal images from an oocyte expressing APPL1-GFP (20 ng/oocyte) and Rab5-RFP (20 ng/oocyte) showing their partial colocalization. Arrows indicate smaller vesicles that are APPL1-positive but Rab5-negative. Scale bar 1 μm. (B) Rendition in 3D of a z-stack of confocal images showing the distribution of APPL1-positive vesicles across the entire stack volume. Vesicles are color-coded with the heat map indicating the colocalization intensity of individual vesicle of both APPL1-GFP and Rab5-RFP. (C) Distribution of APPL1-positive vesicles as a function of vesicle volume and colocalization with Rab5. Colocalization intensity was calculated by the Imaris software for each vesicle within the rendered volume as f=i1*i2 where i1 and i2 are the intensities in the individual channels. Colocalization intensity was normalized to maximal colocalization. We define Rab5-negative vesicles as vesicles where the normalized colocalization in below 0.15, that is 15% of the maximal colocalization. (D) We define VEEs as APPL1-positive and Rab5-negative vesicles, which have an average volume of 0.045 μm3. (E) Normalized colocalization of APPL1 and mPR in VEE endosome (vesicle ≤ 0.045 μm3), EE (vesicles >0.045 μm3) and in the entire vesicle population (All) at time 0 minutes (T0) and at the time point between 2 and 30 minutes when maximal colocalization is observed in different batches of oocytes (Max). The data are normalized to T0 for each vesicle population (mean ± SEM, n = 7). *p < 0.05. Refer to S1 Data file. APPL1, Adapter protein containing Pleckstrin homology domain, Phosphotyrosine binding domain and Leucine zipper motif 1; GFP, green fluorescent protein; ns, not significant; VEE, very early endosome; Rab5, Ras-related protein Rab-5A. (TIF) [file pbio.3000901.s006.tif]
