# Supplementary material for: Membrane progesterone receptor induces meiosis in Xenopus oocytes through endocytosis into signaling endosomes and interaction with APPL1 and Akt2
Source: PLoS Biol. 2020 Nov 2;18(11):e3000901. doi: 10.1371/journal.pbio.3000901 (PMC7660923; doi:10.1371/journal.pbio.3000901)
Supplement: S1 Table — (DOCX) [file pbio.3000901.s007.docx]

**S1 Table**

**List of antibodies, chemicals and reagents used in this study**

| **REAGENT** | **SOURCE** | **IDENTIFIER** |
| --- | --- | --- |
| *Antibodies* |  |  |
| Rabbit polyclonal anti-APPL1 | Toronto Biosciences | Cat# 11130 |
| Rabbit polyclonal anti-GFP used in WB | Living Colors | Cat# 632381 |
| Rabbit polyclonal anti-GFP used in IHC | Abcam | Cat# ab290 |
| Rabbit polyclonal anti-MEK1/2 | Abcam | Cat# 131517 |
| Mouse monclonal anti-SNAP25 | Biolegend | Cat# 836304 |
| Rabbit polyclonal anti-p-plk1 | Santa Cruz biotechnology | Cat# sc-135706 |
| Mouse monoclonal anti-phospho-MAPK | Cell Signaling | Cat# 9106 |
| Rabbit polyclonal anti-phospho-Cdc2 | Cell Signaling | Cat# 9111 |
| Rabbit polyclonal anti-tAKT | Cell Signaling | Cat# 9272S |
| Rabbit polyclonalanti-p-AKTS473 | Cell Signaling | Cat# 9271S |
| Rabbit polyclonalanti-Tubulin | Cell Signaling | Cat# 3873 |
| Goat anti-rabbit IgG-HRP | Jackson ImmunoResearch | Cat# 111-035-144 |
| Goat anti-rabbit IgG- IRDye® 800 | Li-COR | Cat# 925-32211 |
| Goat anti-mouse IgG- IRDye® 680 | Li-COR | Cat# 925-68070 |
| Goat anti-mouse IgG- IRDye® 800 | Li-COR | Cat# 926-32210 |
| Goat anti-rabbit Alexa Fluor 546 | Thermofisher | Cat# A-11030 |
| Mouse monoclonal anti-α-HA | Covance | Cat# MMS-101P |
| Mouse monoclonal anti Cy3-conjugated | Invitrogen | Cat# A10521 |
| *Chemicals and kits* |  |  |
| Progesterone | Sigma-Aldrich | Cat# 200-350-9 |
| Progesterone-BSA conjugate | Bio-Rad | Cat# 7720-0750 |
| OD 02-0 (10- ethenyl-19-norprogesterone (19-CH2P4) | Axon Medchem | Cat# 13258-85-0 |
| Wheat Germ Agglutinin Alexa Fluor^TM^ 633 Conjugate | ThermoFisher | Cat# W21404 |
| Hoechst 33342 | ThermoFisher | Cat# H3570 |
| Pitstop® 2 | Abcam | Cat# ab120687 |
| Pitstop® 2 - negative control | Abcam | Cat# ab120688 |
| Dyngo | Abcam | Cat# ab120689 |
| Dynasore | Abcam | Cat# ab120192 |
| Lipofectamin 2000 | ThermoFisher | Cat# 11668030 |
| Lipofectamine RNAiMAX Transfection Reagent | ThermoFisher | Cat# 13778030 |
| Digitonin | Abcam | Cat# ab141501 |
| Mammalian ProteaseArrest^TM^ | G-Biosciences | Cat# 786-331 |
| PhosphataseArrest^TM^ | G-Biosciences | Cat# 786-451 |
| Calf intestinal phosphatase (CIP) | New England Biolabs | Cat# M0290 |
| µMACS GFP Isolation Kit | Miltenyi Biotech | Cat# 130-091-125 |
| ECL™ Prime Western Blotting Detection Reagent | GE Healthcare Amersham™ | Cat# 45-002-401 |
| QIAGEN LongRange PCR Kit | Qiagen | Cat# 206402 |
| The XL QuikChange mutagenesis kit | Agilent Technologies | Cat# 200516 |
| The mMessage mMachine T7 kit | ThermoFisher | Cat# AM1344 |
| Fast SYBR^TM^ Green Master Mix | ThermoFisher | Cat# 4385616 |
| *siRNAs* |  |  |
| APPL1 siRNA | Origene | Cat# SR419358 |
| AllStars Negative Control siRNA | Qiagen | Cat# SI03650318 |
| *Software* |  |  |
| Image Studio 5.2 | LI-COR Biosciences | [N/A](https://www.licor.com/bio/image-studio/) |
| ImageJ | Schneider et al. 2012 | [N/A](https://www.licor.com/bio/image-studio/) |
| Imaris x64 9.2.1 | Bitplane | [N/A](https://www.licor.com/bio/image-studio/) |
| Prism | GraphPad inc. | [N/A](https://www.licor.com/bio/image-studio/) |
| Illustrator CC | Adobe Systems Inc | [N/A](https://www.licor.com/bio/image-studio/) |
| Photoshop CC | Adobe Systems Inc | [N/A](https://www.licor.com/bio/image-studio/) |
| ZEN 2.3 black | Zeiss | [N/A](https://www.licor.com/bio/image-studio/) |
| Quant Studio Real-Time PCR software.Ink | ThermoFisher | [N/A](https://www.licor.com/bio/image-studio/) |
| 3D Modeling | SWISS-MODEL Workspace / GMQE | [N/A](https://www.licor.com/bio/image-studio/) |
