# Supplementary material for: Membrane progesterone receptor induces meiosis in Xenopus oocytes through endocytosis into signaling endosomes and interaction with APPL1 and Akt2
Source: PLoS Biol. 2020 Nov 2;18(11):e3000901. doi: 10.1371/journal.pbio.3000901 (PMC7660923; doi:10.1371/journal.pbio.3000901)
Supplement: S2 Table — (DOCX) [file pbio.3000901.s008.docx]

**S2 Table**

**List of antisense oligonucleotides and primers used in this study**

| Control antisense | gcgactatacgcgcaatatg |
| --- | --- |
| APPL1 Control sense | agagcgatgtaggggaggaa |
| APPL1.L antisense | ttcctcccctacatcgctct |
| mPR antisense | cggtagtcatggtagtaggg |
| AKT2 antisense | aaatatcgtggtctccatgt |
| mPR-GFP ∆N - ECOR1 F | cgaattcgcatgaagaagcacaatgaatctgtc |
| mPR-GFP ∆N - XhoI R | actcgagttacttgtacagctcgtccatgc |
| mPR-GFP ∆C - F | atggtgagcaagggcgaggagctg |
| mPR-GFP ∆C - R | ccgtgcataaacggcagtgaaggtgc |
| HA-XmPR-GFP - BamHI-HA F | ctcggatccacccatgtacccatacgatgttccagattacgctactaccggaatc |
| HA-XmPR-GFP - XbaI R | cggtctagaattaaagttcttttctggccaacttttccttgttcttctccgtgc |
| mPR-GFP HA - BamHI F | ctcggatccacccatgactaccgcaatccttgaatg |
| mPR-GFP HA - HA-XbaI R | ctttctagaattaatctggaacatcgtatgggtaaagttcttttctggc |
| mPR H129A – F | tctcacatgcagcatcctggctgctctgctccagtc |
| mPR H129A – R | gactggagcagagcagccaggatgctgcatgtgaga |
| mPR D146A – F | cattacaccttctacttcatagcctatgtaggagtaagcacttac |
| mPR D146A – R | gtaagtgcttactcctacataggctatgaagtagaaggtgtaatg |
| mPR H281/285A – F | cgattttatagggcacggtgcccagattttcgctgtgtttctggg cctct |
| mPR H281/285A - R | agaggcccagaaacacagcgaaaatctgggcaccgtgcccta taaaatcg |
| Xenopus mPRβ – F | cccgttgtccaccggatagt |
| Xenopus mPRβ – R | ggtgaccgtgccctataaaa |
| Xenopus APPL1.L – F | gagcgtcggataaacagaaag |
| Xenopus APPL1.L – R | cgactctcgctgcttcttct |
| Xenopus APPL1.S – F | aggccgctctctgtctgtta |
| Xenopus APPL1.S – R | aaactgtccctcagcattgg |
| Xenopus AKT1 – F | cccgctactttctcctcaaa |
| Xenopus AKT1 – R | ggtcgttctgtcttcatcagc |
| Xenopus AKT2 – F | tcctaatgatgcccaagagg |
| Xenopus AKT2 – R | agggcgctgatctgattcta |
| Xenopus ODC – F | gccattgtgaagactctctccattc |
| Xenopus ODC – R | ttcgggtgattccttgccac |
