# Supplementary material for: Membrane progesterone receptor induces meiosis in Xenopus oocytes through endocytosis into signaling endosomes and interaction with APPL1 and Akt2
Source: PLoS Biol. 2020 Nov 2;18(11):e3000901. doi: 10.1371/journal.pbio.3000901 (PMC7660923; doi:10.1371/journal.pbio.3000901)
Supplement: S3 Table — The most effective antisense oligos that were used for the knockdown experiments are bolded. Akt2, protein kinase B; APPL1, Adapter protein containing Pleckstrin homology domain, Phosphotyrosine binding domain and Leucine zipper motif 1; mPR, membrane progesterone receptor. (DOCX) [file pbio.3000901.s009.docx]

**S3 Table**

**List of mPR/APPL1/AKT2 antisense oligonucleotides tested in this study.**

The most effective antisense oligos that were used for the knockdown experiments are bolded.

| **Primer** | **Sequences (5' -> 3')** |
| --- | --- |
| **Paqr8 (mPR)** |  |
| **161** | **CGGTAGTCATGGTAGTAGGG** |
| 204 | TGCTGGAAACTGATGGAGAG |
| 467 | CATTGAGAGAAAGAGAACCG |
| 514 | ATAGGTGAGCGAGGATAGCA |
| 942 | GGGCAGGAAAAGAAATAGGC |
|  |  |
| **APPL1** |  |
| 249 | CAGGAGTTTGGAGGTCAGGT |
| 845 | GGCAAGTACAATGGCTCACT |
| 883 | GGTTGCGATTCACAGGGAGT |
| 921 | CTTGTTGCGTATGTTGAGGT |
| 1416 | GAACTGGATGGGAGTGTCTG |
| **2148** | **TTCCTCCCCTACATCGCTCT** |
|  |  |
| **AKT2** |  |
| 910 | GCTCCATCTGTGATGCCTTC |
| 835 | TTCTCCAGCTTGATGTCTCT |
| **61 (AS1)** | **AAATATCGTGGTCTCCATGT** |
| 309 | TGGCCACTGTCTGGATGGCA |
| **1441 (AS2)** | **TACTCCCGTATGCTTGCAGA** |
