# Supplementary material for: Membrane progesterone receptor induces meiosis in Xenopus oocytes through endocytosis into signaling endosomes and interaction with APPL1 and Akt2
Source: PLoS Biol. 2020 Nov 2;18(11):e3000901. doi: 10.1371/journal.pbio.3000901 (PMC7660923; doi:10.1371/journal.pbio.3000901)

Full Western Blot: Fig 1G

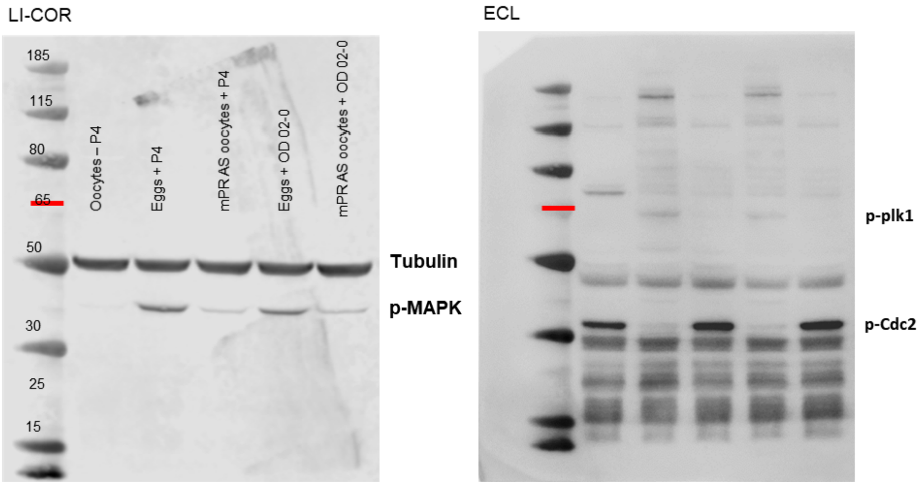

Full Western Blot: Fig 2A

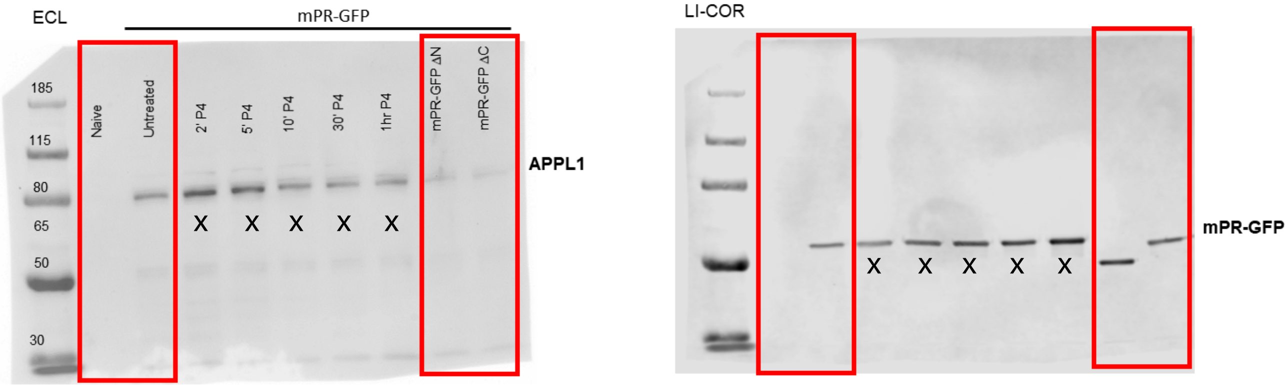

Full Western Blot: Fig 2C

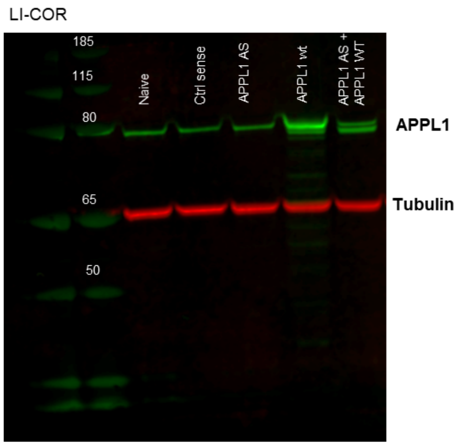

Full Western Blot: Fig 2G

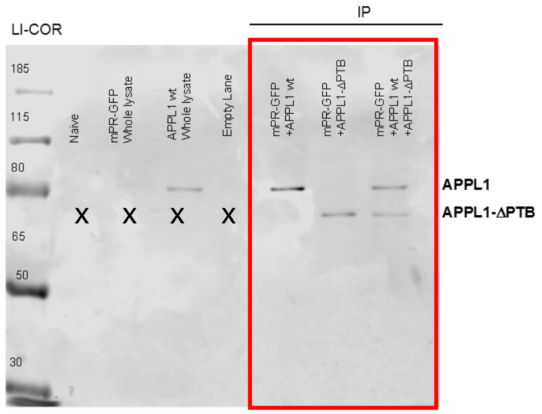

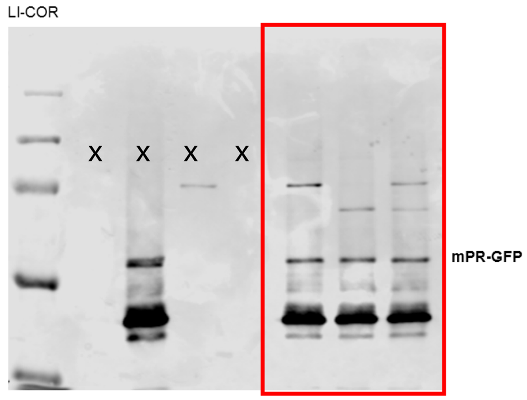

Full Western Blot: Fig 2I

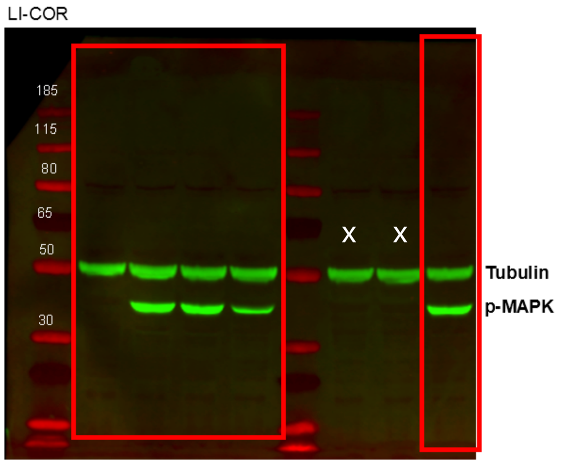

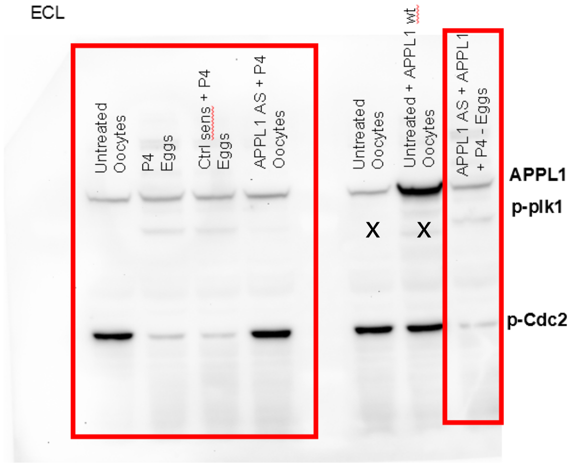

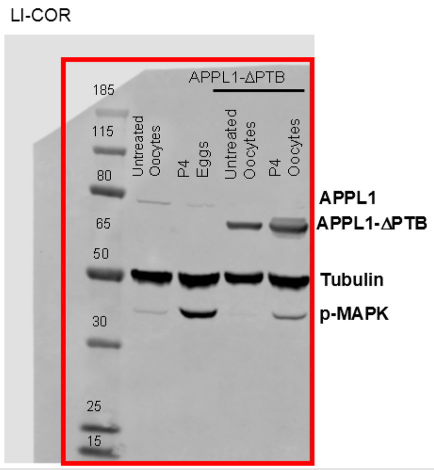

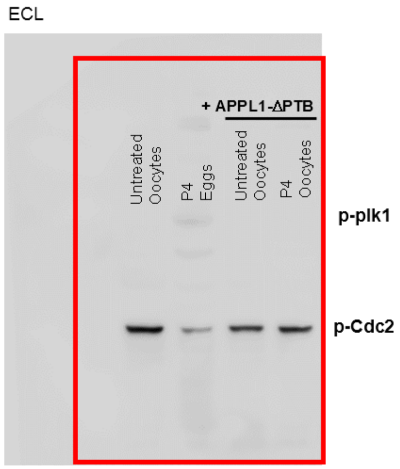

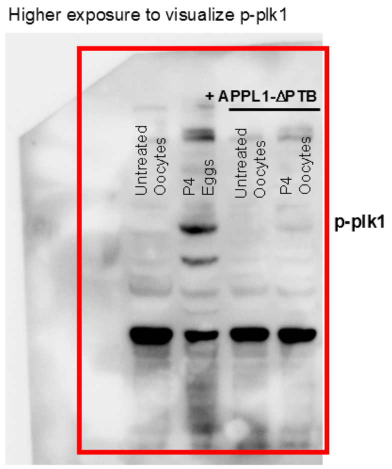

Full Western Blot: Fig 2M

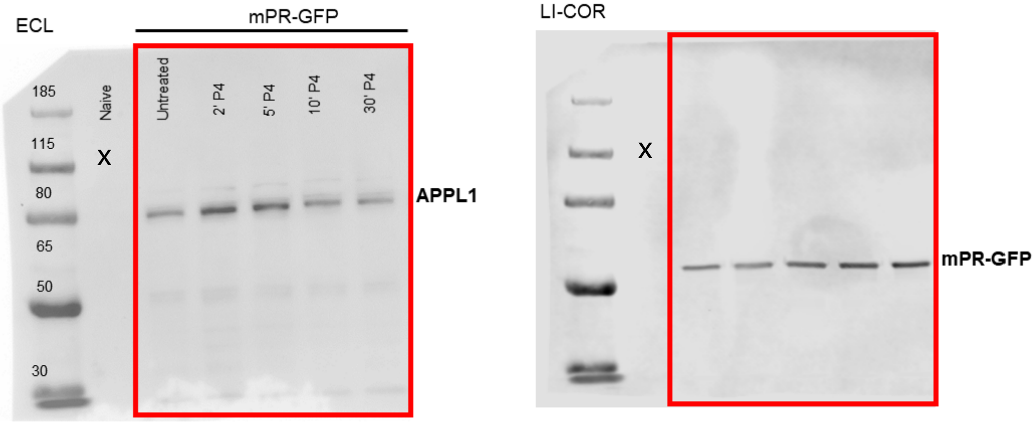

Full Western Blot: Fig 3B

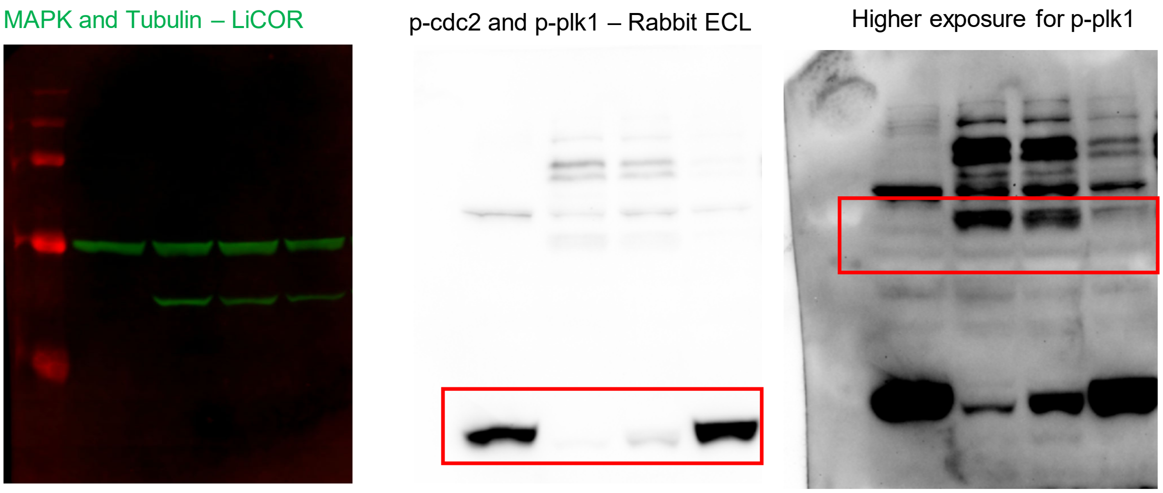

Full Western Blot: Fig 3D

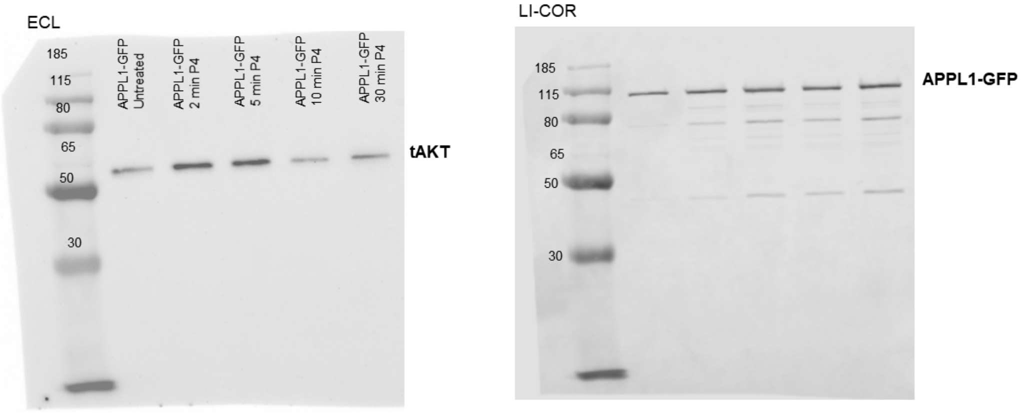

Full Western Blot: Fig 3F

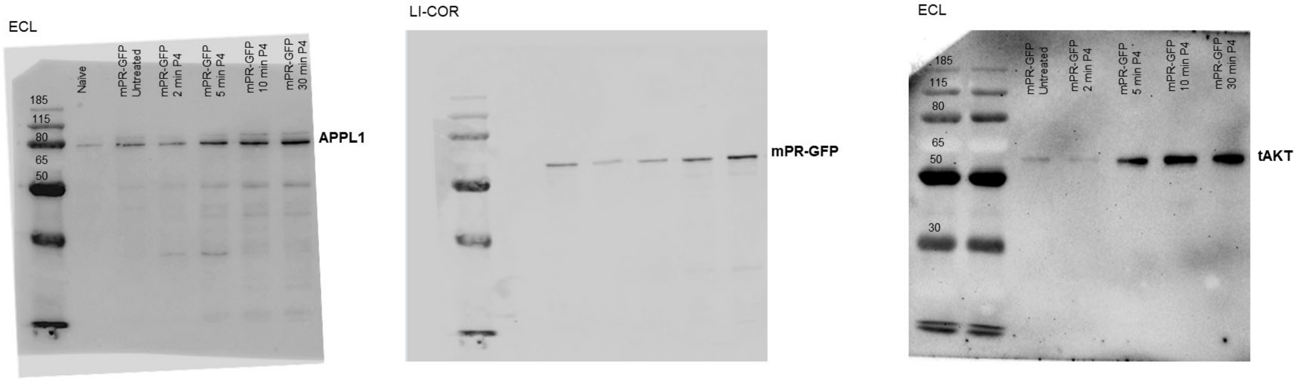

Full Western Blot: Fig 3I

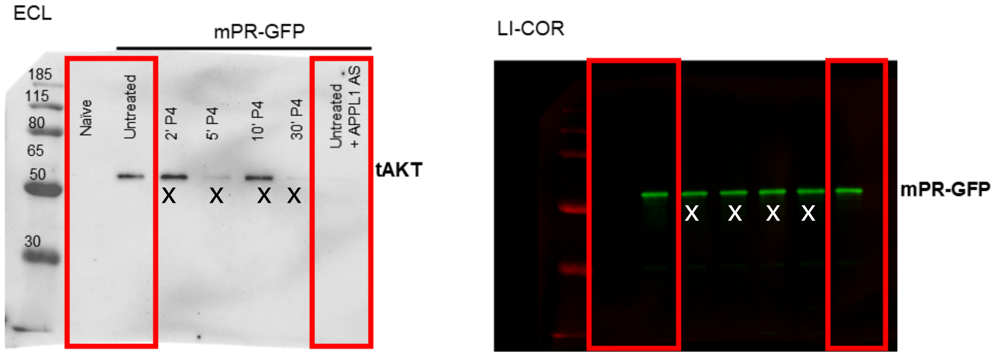

Full Western Blot: Fig 3J

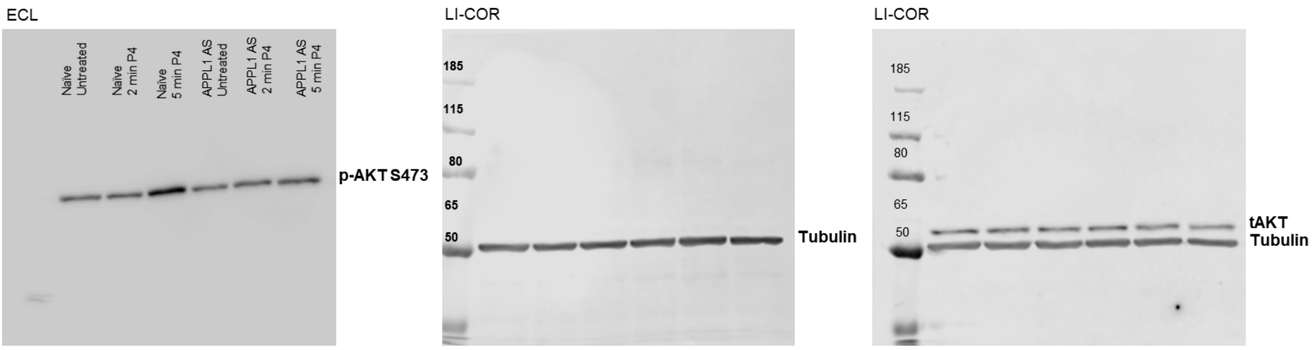

Full Western Blot: Fig 4B

LI-COR

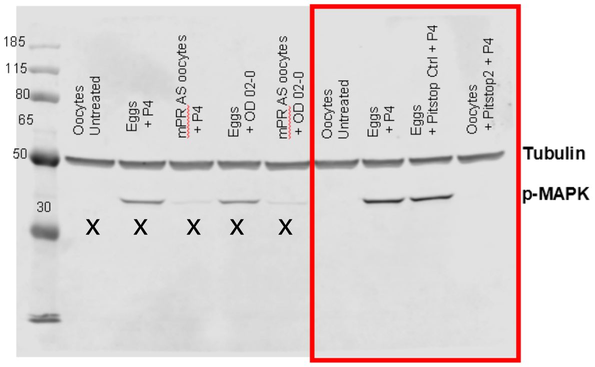

ECL

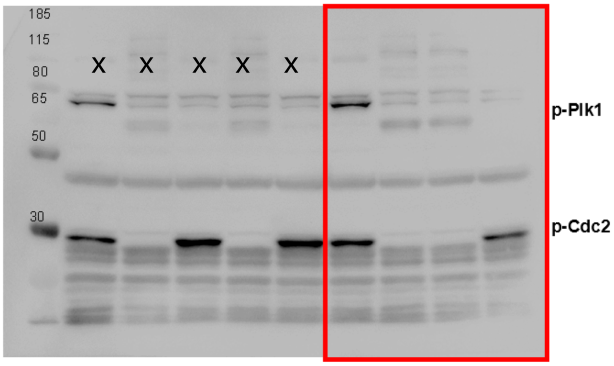

Full Western Blot: S1F Fig

Tubulin LI-COR

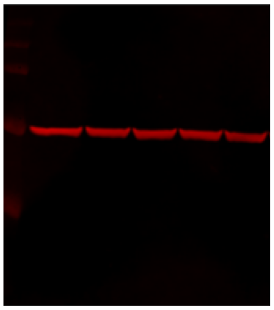

GFP ECL

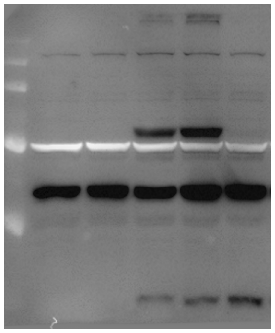

Full Western Blot: S3B Fig

ECL

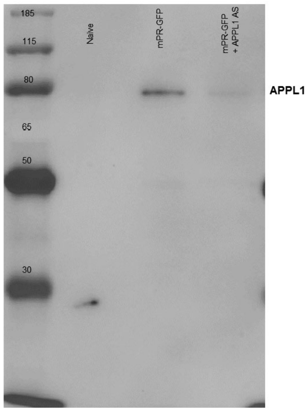

LI-COR

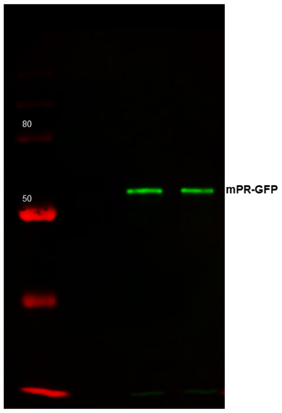

Full Western Blot: S3D Fig

LI-COR

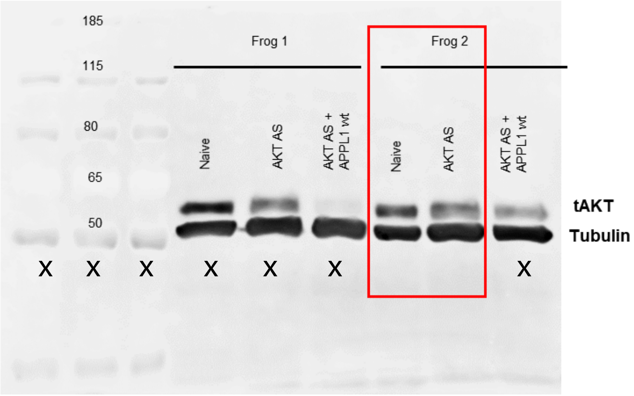

Full Western Blot: S3E Fig

ECL (Akt), LI-COR (Tub)

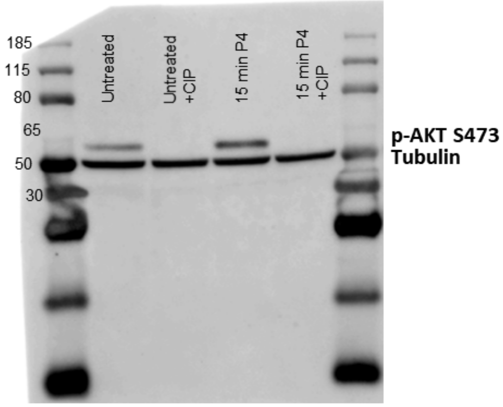

LI-COR

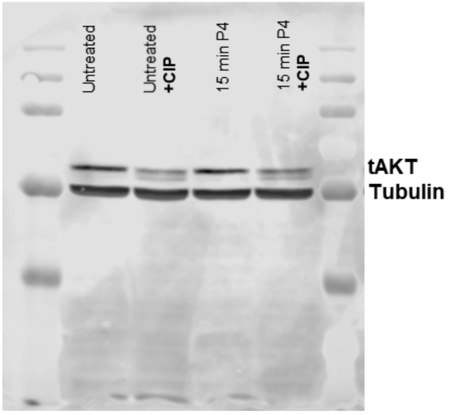

Full Western Blot: S5A Fig

LI-COR

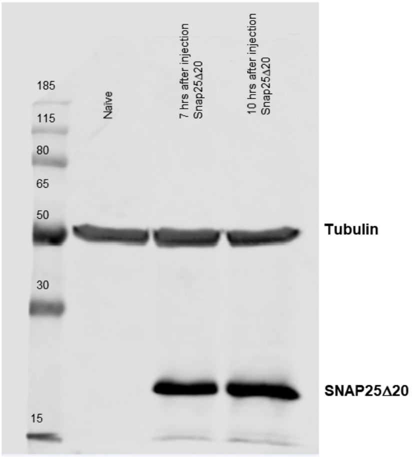

Supplement: S1 raw images — (PDF) [file pbio.3000901.s011.pdf]
